# Genome-wide methylation analysis identifies genes silenced in non-seminoma cell lines

**DOI:** 10.1038/npjgenmed.2015.9

**Published:** 2016-01-13

**Authors:** Dzul Azri Mohamed Noor, Jennie N Jeyapalan, Safiah Alhazmi, Matthew Carr, Benjamin Squibb, Claire Wallace, Christopher Tan, Martin Cusack, Jaime Hughes, Tom Reader, Janet Shipley, Denise Sheer, Paul J Scotting

**Affiliations:** 1 School of Life Sciences, University of Nottingham, Nottingham, UK; 2 School of Pharmaceutical Sciences, Universiti Sains Malaysia, Pulau Pinang, Malaysia; 3 The Blizard Institute, Barts and The London School of Medicine and Dentistry, Queen Mary, University of London, London, UK; 4 Biology Department, Faculty of Science, King Abdulaziz University, Jeddah, Saudi Arabia; 5 Divisions of Molecular Pathology and Cancer Therapeutics, The Institute of Cancer Research, London, UK

## Abstract

Silencing of genes by DNA methylation is a common phenomenon in many types of cancer. However, the genome-wide effect of DNA methylation on gene expression has been analysed in relatively few cancers. Germ cell tumours (GCTs) are a complex group of malignancies. They are unique in developing from a pluripotent progenitor cell. Previous analyses have suggested that non-seminomas exhibit much higher levels of DNA methylation than seminomas. The genomic targets that are methylated, the extent to which this results in gene silencing and the identity of the silenced genes most likely to play a role in the tumours’ biology have not yet been established. In this study, genome-wide methylation and expression analysis of GCT cell lines was combined with gene expression data from primary tumours to address this question. Genome methylation was analysed using the Illumina infinium HumanMethylome450 bead chip system and gene expression was analysed using Affymetrix GeneChip Human Genome U133 Plus 2.0 arrays. Regulation by methylation was confirmed by demethylation using 5-aza-2-deoxycytidine and reverse transcription–quantitative PCR. Large differences in the level of methylation of the CpG islands of individual genes between tumour cell lines correlated well with differential gene expression. Treatment of non-seminoma cells with 5-aza-2-deoxycytidine verified that methylation of all genes tested played a role in their silencing in yolk sac tumour cells and many of these genes were also differentially expressed in primary tumours. Genes silenced by methylation in the various GCT cell lines were identified. Several pluripotency-associated genes were identified as a major functional group of silenced genes.

## Introduction

Promoter hypermethylation of many different tumour suppressor genes is seen in a wide range of cancers.^1,2^ This has been assumed, though only occasionally demonstrated, to silence the expression of those genes. The term ‘methylator phenotype’ or CpG island methylator phenotype has been coined to describe subgroups of cancers, such as some colon tumours and gliomas, that exhibit particularly high levels of methylation of a consistent subset of genes, usually in and around their CpG islands.^3–7^

Testicular germ cell tumours (TGCTs) are the most common malignancy of young men. Despite high cure rates in response to platinum-based chemotherapy, they still represent a fatal disease in a minority of patients presenting with disseminated disease^8,9^ and the prognosis in children is much worse than in adults.^10^ GCTs are an exceptional group of tumours in many respects. They are the only class of cancer that arises from a pluripotent progenitor cell (the germ cell progenitor, PGC) and that cell exhibits profoundly different DNA methylation characteristics to all somatic cell types. They present as several remarkably varied histological phenotypes classified as seminomatous or non-seminomatous. Seminomatous tumours (called seminomas in the testes, dysgerminomas in the ovary and germinomas in extragonadal sites) exhibit a relatively uniform histology with a similarity to germ cell progenitors. Non-seminomatous tumours, such as yolk sac tumours (YSTs) and embryonal carcinomas (EC), tend to be more aggressive and resistant to therapy than seminomatous tumours,^8,9,11^ especially in intracranial cases seen in children.^10^

Despite frequently having already metastasised at presentation, most TGCTs are exceptionally chemosensitive. Their progression from Intratubular Germ Cell Neoplasia, Unspecified (ICGNU) gives rise to seminoma or to the various non-seminomas. The more aggressive and chemoresistant non-seminomas can arise from seminoma, even within the same tumour^12^ or as a recurrence after treatment.^13^ There is some evidence that progression to non-seminomas involves a dramatic increase in DNA methylation.^14,15^ Since all forms of GCT are believed to progress from ICGNU, which, like germ cell progenitors, is hypomethylated, methylation must be an event associated with their progression rather than tumour initiation.^16^

Two recent studies of the global methylation of paediatric GCTs demonstrated the hypermethylation of many candidate tumour suppressor genes.^14,15^ Although these showed a dramatic difference in methylation between GCT subtypes, with seminomas showing much less methylation than non-seminomas, they could not identify, in an unbiased manner, those genes that were silenced by methylation. A critical question, therefore, is the extent to which methylation is linked to gene silencing and how the position of that methylation within the genes relates to this.

In this study, we set out to analyse the relationship between DNA methylation of different genes and gene elements and consequent gene silencing. For this purpose, we needed to rely on cell lines because they provide a more homogenous system (as compared with the heterogeneity of primary tumour samples) and where the causative role of DNA methylation in gene silencing can be tested. Two recent studies have been published that analysed global DNA methylation in GCT cell lines. Rijlarsdaam *et al.*^17^ analysed methylation in cell lines derived from multiple types of GCT but they did not determine the relationship of this methylation to gene expression, while van der Zwan *et al.*^18^ analysed both methylation and gene expression, but only in seminoma versus EC cell lines. Here, the Illumina infinium HumanMethylome450 bead chip system, which surveys over 99% of RefSeq genes with an average of 17 CpG sites per gene was used. To gain a comprehensive view of the correlation between methylation and gene expression, these same cell lines were analysed using Affymetrix expression arrays. Finally, key genes identified were tested to determine if they were activated by demethylation, confirming that DNA methylation was indeed playing a role in their reduced expression. These data were also compared with gene expression in a cohort of primary GCT samples using the same array platform. These data confirm that the cell lines derived from different histological subtypes of non-seminoma exhibit much greater gene-associated methylation than the seminoma cells and identify a group of pluripotency-associated genes, which are silenced in the YST as compared with the seminoma cell line.

## Results

### Relationships between genes methylated in different GCT subtypes

**Figure 1:**
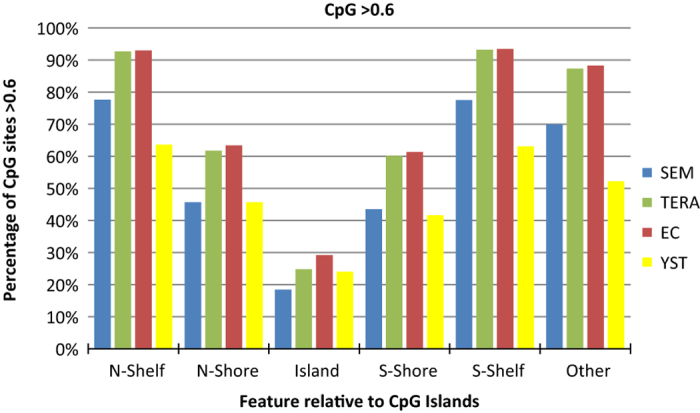
Percentage of CpGs methylated (*β*-value ⩾0.6) described relative to CpG islands. The 450 K arrays provide a quantitative reading (*β*-value) from 0 (unmethylated) to 1 (completely methylated) for individual CpG sites, each described in relation to the closest gene and the nearest CpG island. These are annotated as island (a region of at least 500 bp, with >55% GC and an observed-to-expected CpG ratio >0.65), shore (regions 2 kb either side of an island), shelf (regions 2 kb outside of the shores) or ‘other’, also referred to as ‘open sea’.

DNA methylation was analysed in four adult GCT-derived cell lines (TCam-2, GCT44, GCT27 and NT2D1, subsequently referred to as Seminoma, YST, EC and Teratoma cell lines, respectively) on a genome-wide scale using the Infinium HumanMethylome450 array chip (University of London, London, UK; See Supplementary Dataset 1 and 2). In all cell lines, the lowest level of methylation was concentrated in CpG islands (Figure 1).

All three non-seminoma cell lines showed higher numbers of methylated island CpGs (*β*-value ⩾0.6) than the seminoma cell line. For the EC and teratoma cell lines, a similar degree of difference to seminoma was also seen in all other regions (shores, shelves and ‘open sea’), whereas for the YST cells this difference decreased in the shores and the number of methylated CpGs in shelves and open sea was lower than that in the seminoma cells where many more were unmethylated (*β*-value <0.3) (Figure 1).

**Figure 2:**
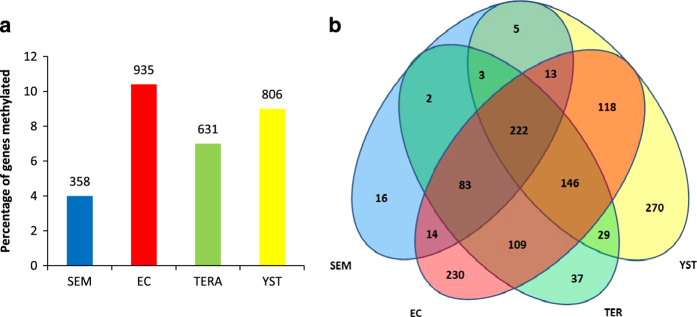
Gene methylation based on average methylation across CpG islands in the TSS region. A gene was considered to be methylated when the average *β*-value across all CpGs analysed in CpG island(s) located near to the gene’s TSS (within 1,500 bp upstream of the TSS or within the 5′ UTR/first exon) was >0.6. (**a**) Number of genes methylated (average *β*-value ⩾0.6) is represented as a percentage of all genes associated with a CpG island. Values above bars indicate number of gene. (**b**) Venn diagram representing the overlap between genes methylated in each cell line. The gene count of each section is indicated.

We next set out to identify the specific genes where CpG islands were differentially methylated between seminoma and non-seminoma cell lines. We chose to initially select those genes that were methylated across CpG islands near to the TSS (CpG islands exhibiting an average methylation *β*-value ⩾0.6 were recorded as methylated). This analysis showed that the EC cell line had the highest number of genes with TSS-associated methylated CpG islands, followed by YST, teratoma and seminoma cells (Figure 2a).

To establish similarities and differences between the cell lines, the overlap in the lists of genes methylated in each cell type were identified (Figure 2b). Genes methylated in seminoma cells are largely a subset of those methylated in EC and teratoma cells. Indeed, 94% of genes (337/358) methylated in the seminoma cell line are also methylated in EC and/or teratoma cells with 62% of these genes being methylated in all three non-seminoma subtypes (Figure 2b). The population of genes methylated in the YST cell line appears to be more strikingly different. The YST cell line exhibited the highest number of uniquely methylated genes relative to the total number of methylated genes (270/806, Figure 2b). By comparison, only 16 genes were methylated uniquely in the seminoma cell line.

### Transcriptome analysis reveals that over 50% of differentially methylated genes show a corresponding difference in gene expression

The best-documented mechanism for a gene methylation event to contribute to the biology of a cell is through altering that gene’s expression. We therefore set out to determine to what extent the gene methylation events described above were reflected in silencing of these genes.

**Figure 3:**
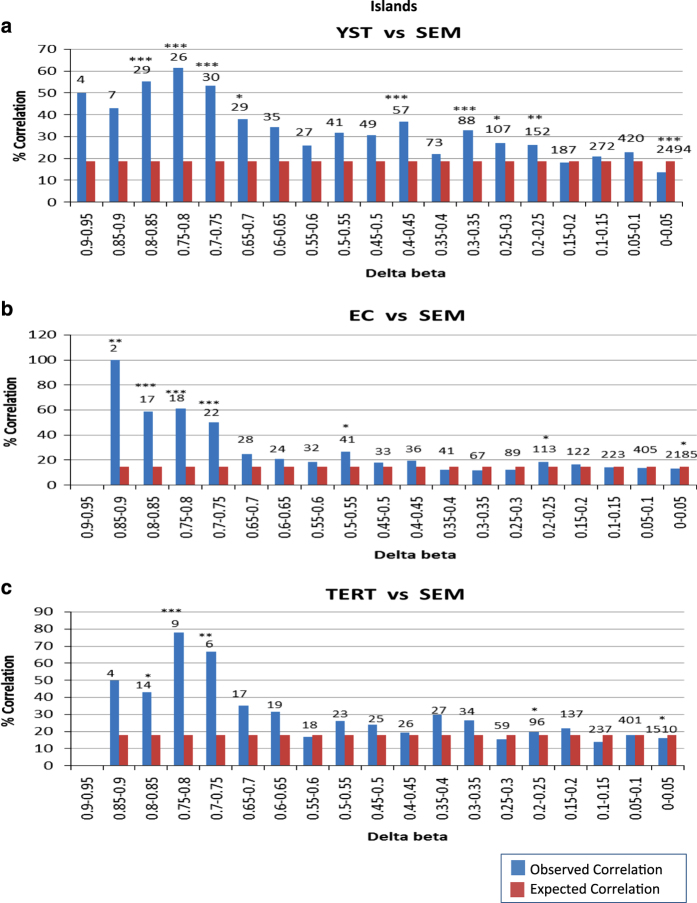
Correlation between differential expression of over twofold and different degrees of differential CpG island methylation between seminoma and non-seminomas. Histograms showing the observed (blue) and expected (red) percentage correlation for each Δ*β*-value range for TCAM2 (seminoma) versus GCT44 (YST) (**a**), GCT27 (EC) (**b**) and NT2D1 (teratoma) (**c**). A Δ*β*-value of >0.65 or 0.7 consistently correlated significantly with a difference in expression of greater than twofold. While some lower Δ*β*-value categories also show significant association, statistical significance was reached at much smaller percentage levels of association due to a larger number of genes in those differential methylation ranges (see Figure 2 for details) (for example, comparing GCT44 to TCAM2, 152 genes exhibit Δ*β*-values between 0.2–0.25 with only 26% show a correlation with decreased expression (over a random expected association of 18%, but this achieves a *P* value <0.01). On the other hand, due to the small numbers of genes exhibiting some of the highest differential methylation values, these were not significantly associated with differences in expression, often despite a large percentage levels of association (for example, comparing GCT44 to TCAM2, seven genes exhibit a Δ*β*-values between 0.85 and 0.9 of which three (43%) show a correlation with decreased expression, but this does not achieve a significant *P* value). Significance of the *Χ*^2^-tests of association are shown (*P<0.05, **P<0.01, ***P<0.001) and the total number of genes in each category is displayed above the bar.

RNA was isolated from each of the cell lines and subjected to gene expression analysis using Affymetrix U133 plus 2 chips (the University of Nottingham, Nottingham, UK; See Supplementary Dataset 3). The data were analysed to assess the relationship between CpG methylation and gene expression. In particular, we asked if high methylation correlated with low expression (see Supplementary Dataset 4). The degree of correlation between the level of differential methylation of the various gene elements (regarding islands, shores or shelves) and inverse gene expression was analysed pairwise between cell lines. We divided gene elements into categories of differential methylation at average Δ*β* intervals of 0.05 across those elements (see Table 1). The lowest category (Δ*β*=0 to 0.05) corresponds to a large group of genes, which showed similar levels of methylation in seminoma and non-seminoma lines, while the highest category (Δ*β*=0.9 to 0.95) corresponds to genes that showed the greatest increase in methylation in the seminoma line relative to non-seminoma lines. For each category, we calculated the expected frequency of genes showing more than twofold differential expression under the null hypothesis that lower gene expression does not correlate with methylation. For each category of methylation, we then applied a Pearson’s *Χ*^2^-test to determine whether the observed frequency of differential expressed genes was greater than expected by chance. The aim of this approach was to provide us with an objective basis for the selection of genes where a correlation between differential methylation and differential expression was likely to be of biological significance. The resulting data (for CpG islands comparing the non-seminoma and seminoma cell lines) are shown in Table 1. These data were then used to generate graphs showing the percentage of genes with more than twofold differential expression for each Δ*β*-value category (Figure 3, Supplementary Figure S1).

Comparing the YST and seminoma cell lines, there was a substantial (greater than two times the value expected at random) and significant correlation between lower expression in YST cells and a difference in methylation >0.65 Δ*β*-value (Figure 3a). For this reason, all further analyses excluded genes that were differentially expressed but where differential methylation was <0.65, since for any given gene a correlation with a lower level of differential methylation is more likely to simply reflect a random association. Similar comparison of EC and teratoma cell lines to the seminoma cell line found that a significant and substantial association between methylation and silencing of expression was reached at an average Δ*β*-value of over 0.7 (Figures 3b,c). For all non-seminoma cell lines, islands showed a stronger correlation with reduced expression than methylation of shores or any other regions (Figure 3, Supplementary Figure S1).

**Figure 4:**
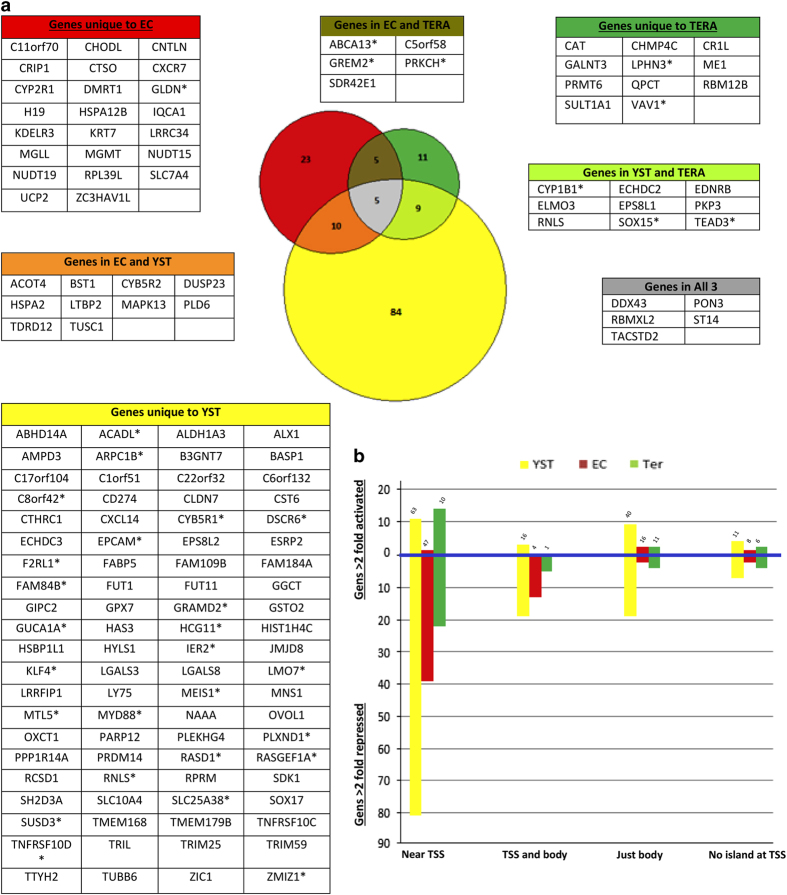
Genes differentially methylated and differentially expressed between seminoma and non-seminoma cell lines. (**a**) Tables show genes expressed at higher levels in seminoma than in non-seminoma cell lines (⩾2-fold difference in expression from microarray data), that are also significantly more methylated (Δ*β* ⩾0.7 for EC and teratoma, ⩾0.65 for YST) in the non-seminoma lines. Differential methylation was associated with CpG islands near to a TSS, except for those genes marked with an asterisk where methylation was across CpG islands in the body of the gene. These same genes are shown as numbers in the various overlapping cell types (colours match tables) in the central Venn diagram. (**b**) Graph showing numbers of genes showing differential expression between seminoma and various non-seminoma cell lines grouped according to exhibiting differential methylation in body and TSS regions (all genes included exhibit greater methylation (Δ*β* ⩾0.65) in a non-seminoma versus the seminoma cell line across the island. Numbers above each bar indicate the number of genes that were differentially methylated but showed less than a twofold difference in expression between the cell lines.

Based on the Δ*β*-value thresholds established above those genes expressed in seminoma but not in the non-seminoma cell lines, where reciprocal differential methylation of CpG island near the TSS was implicated in their silencing in non-seminoma cells, were identified (Figures 3 and 4a). Among genes differentially methylated at a TSS-associated CpG island between non-seminoma and seminoma cell lines (for which expression data were available) about half showed a correlating decreased expression in the various non-seminoma cell lines. It was notable that the genes identified in this way feature high among the most differentially expressed genes between seminoma and the various non-seminoma cell lines. Of the top 10 most differentially expressed genes (for which we have methylation data) in the non-seminomas, 4 are differentially methylated in EC cells, 3 in YSTs and 2 in teratoma cells. Thus it seems that differential methylation could play a substantial role in the differential gene expression between seminoma and non-seminoma cells.

### Strong correlation between methylation of islands in gene bodies and gene silencing

In previous studies, methylation in gene bodies has been associated with active genes.^19,20^ However, using the same cut-off of 0.65 Δ*β*-value for differential methylation and a twofold difference in gene expression described above, we found that increased methylation in body CpG islands was more strongly associated with gene silencing than activation (Figure 4b). In the YST cells line, 45 out of 128 genes exhibiting increased methylation of body CpG islands compared with the seminoma cells showed a correlating twofold or greater decrease in expression and were only rarely associated with gene activation (Figure 4b). A similar relationship was seen in the EC and teratoma cells (Figure 4b). Although many of these genes also exhibited methylation of a CpG island in the region of the promoter, even for genes with a TSS-associated CpG island that was not differentially methylated, the body CpG island methylation was still more strongly associated with silent rather than active genes (Figure 4b). In total, 34 genes for which body CpG island methylation correlated with silencing of expression either lacked a promoter-associated island or these other islands were not differentially methylated. These genes were therefore included in subsequent analysis (asterisk in Tables in Figure 4a).

### Validation of genes silenced by methylation

**Figure 5:**
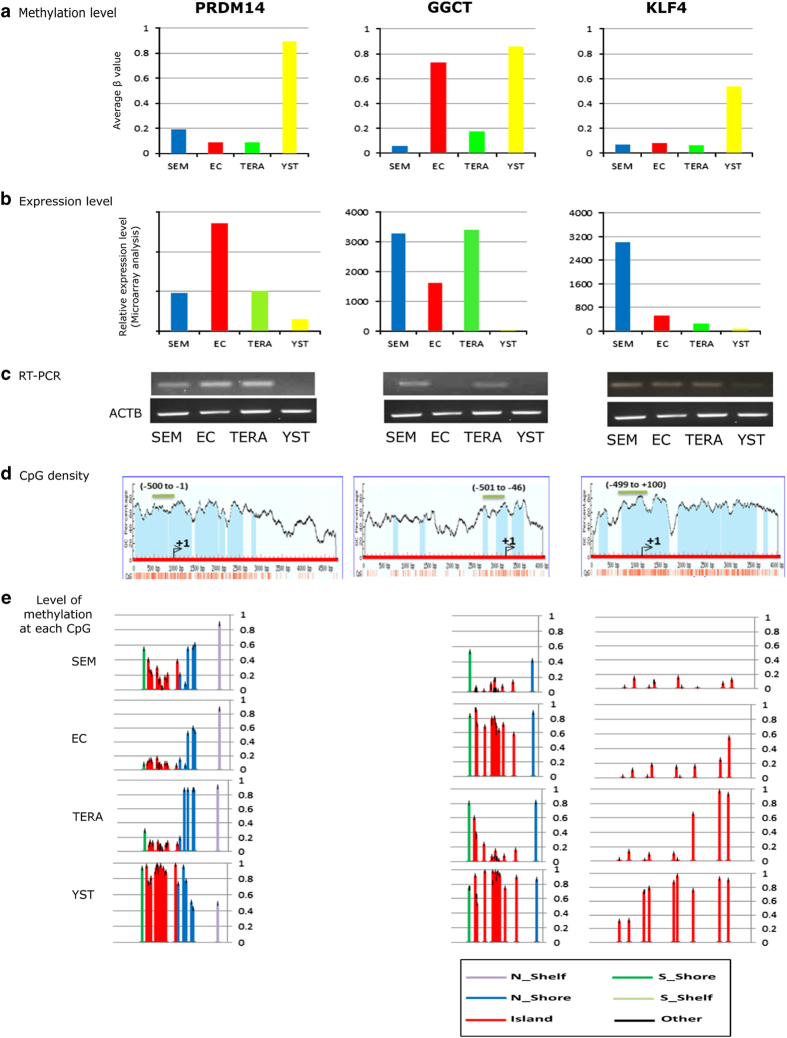
Graphical representation of selected methylated genes and validation of differential expression. For each gene, panels show (**a**) graph of average methylation across CpG islands in the four cell lines, (**b**) level of expression from array analysis (‘Relative expression level (microarray analysis)’ represents the signal relative to control probes), (**c**) RT–PCR analysis, (**d**) graph of CpG density across the gene structure (green bar indicates position of the promoter) and (**e**) level of methylation at each CpG included in 450 K chips relative to CpG features in **d**. Promoter prediction was achieved using the Eukaryotic Promoter Database (http://epd.vital-it.ch) for identification of transcription start sites (TSSs) together with a review of the literature for each gene; gene sequences were submitted to the MethPrimer programme (http://www.urogene.org/methprimer/) to identify CpG sites and islands. Blue vertical bars represent CpG islands, red vertical bars below graphs represent CpG sites.

The expression of a subset of the above genes was assessed using reverse transcription–PCR (RT–PCR). This confirmed the results of the Affymetrix expression arrays for all 17 genes analysed (Figure 5 and see Supplementary Figure S2). The positions of all the CpGs analysed within each gene were also characterized with reference to the gene structure and all CpGs within the gene (some of which were not included on the methylation array). This verified that in these 17 genes, the differences in average methylation across CpGs annotated as islands did reflect multiple CpGs and that these differences were quite consistent across the whole or large parts of each of those islands.

**Figure 6:**
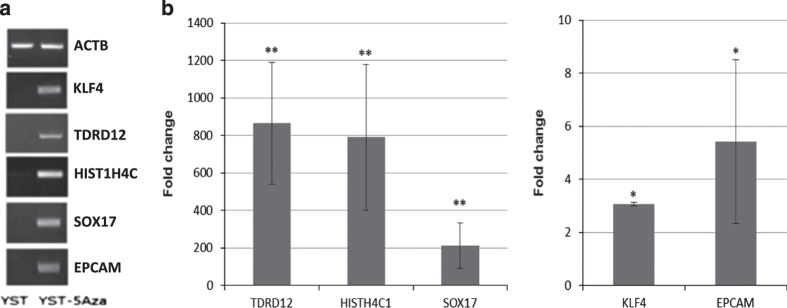
RT–PCR analysis of gene expression for selected genes in YSTs following treatment with 5-deoxyazacytidine. (**a**) Gel image showing activation of five genes following treatment with 5-deoxyazacytidine as compared with control ACTB. (**b**) RT–qPCR data for the same experiments. *P⩽0.01; **P⩽0.001.

A particular reason for using cell lines in this study was that it allowed us to confirm the role of DNA methylation in regulating expression of the genes identified. We therefore tested whether these genes would be re-expressed if demethylated. YST cells were treated for 2 days with 5-aza-2-deoxycytidine and then expression of five of the same 17 genes was reexamined by RT–PCR and RT–quantitative PCR (RT–qPCR). This showed that all five genes were activated by 5-aza-2-deoxycytidine (Figure 6).

### Aberrant gene methylation most likely represents gain of methylation in non-seminoma cells

To determine which methylation events in the cell lines were likely to be aberrant cancer-related events, methylation of the key genes identified above (Figure 4) was compared with a series of control sets of Infinium HumanMethylome450 array methylation data from normal tissues (See Supplementary Dataset 5).

Almost all of the genes identified as methylated in non-seminoma cell lines but unmethylated in seminoma cells were also unmethylated in all control samples. Two striking exceptions to this were the genes DDX43 and TDRD12. DDX43 was heavily methylated in all samples other than seminoma while TDRD12 was methylated in all samples except seminoma and teratoma. Hence, the heavy methylation of these two genes in all control samples implies the difference between GCTs is due to unusual hypomethylation in seminoma (and teratoma for TDRD12).

### Comparison of two data sets comparing seminoma to EC cell lines

Van der Zwan *et al.*^18^ recently using the same 450k methylation chips and Affymetrix expression platforms to compare the same seminoma cell line (TCam-2) with a different EC cell line (NCCIT).^18^ We reanalysed the data of van der Zwan *et al.*^18^ using the same pipeline described above (see Supplementary Datasets 6 and 7; S3 and 4 Tables). Strikingly, 63% of the genes that were differentially methylated in the study of van der Zwan *et al.*^18^ were also differentially methylated in our study; 59% of the differentially expressed genes were shared, and of the 7 genes that were both differentially methylated and differentially expressed (with the methylation correlating with gene silencing) 3 of these (HSPA2, PON3 and TACSTD2) were among the 43 genes in this category in our study. These correlations are highly significant (binomial test *P*<1×10^−7^). These data show that the gene methylation and expression events are remarkably consistent between independent EC cell lines.

### Identification of genes for which methylation is most likely to be of biological significance

To determine if the differences in expression between seminoma and non-seminoma cell lines might be a more general feature of GCTs, we made use of the Affymetrix expression data of Korkola *et al.*^21^ from a cohort of adult TGCTs and Palmer *et al.*^22^ from a cohort of paediatric seminomas and YSTs from many different anatomical locations. Although, such banks of tumour samples would be expected to differ substantially from the gene expression seen in the clonal cell lines used here, we reasoned that genes identified by this comparison, where the role of these genes might be conserved in many GCT samples analysed, would be those events of greatest importance generally in GCT biology.

Comparing seminoma to YST samples, the overlap in genes differentially expressed in these data sets was highly significant (Supplementary Figure S5). Among the genes represented in all three studies, any 2 studies shared ~500–700 genes that were differentially expressed and of these 339 genes were differentially expressed in all 3datasets. Of the 72 genes shown to be both differentially methylated and differentially expressed between the YST and seminoma cell lines in this study (Figure 4), 23 were expressed at higher levels in primary seminomatous tumours than in YSTs (>1.5-fold) in either the Palmer *et al.*^22^ or Korkola *et al.*^21^ data sets with 11 being differentially expressed in both studies (Table 2A). Such a high proportion of differentially expressed genes common to the cell lines and the primary tumours is highly significant (binomial test: *P*<10^−30^) strongly suggesting that the cell lines provide a good surrogate system for studying gene expression in this tumour type. It is also striking that several of these genes encode proteins that are involved in pluripotency and the inhibition of differentiation (see Discussion below). Comparison of the differentially expressed genes between our data and that of Korkola, *et al.* ^21^ for EC and teratoma cell lines also showed much weaker overlap of the genes differentially expressed, although this was still significant for the EC cell data. However, it is noteworthy that three of the five genes methylated and differentially expressed in all three non-seminoma cell lines (DDX43, PON3 and RBMXL2) were also differentially expressed in all three tumour types in the data of Korkola *et al.*^21^ (Tables 2B,C).

## Discussion

This study describes the first comprehensive analysis of global methylation and its relationship to gene expression in cell lines of the major subtypes of GCTs. Of the 7,244 genes that produced reliable signals in the expression array dataset, ~2% (147) of the genes showed correlating differential methylation and expression in our data, of which we were able to identify 23 genes with similar differential expression in cohorts of primary tumour samples.

### Global differences in methylation between GCT cell lines

This study confirms the suggestion from analysis of fewer genes that non-seminoma cells exhibit very high levels of gene methylation as compared with seminoma cells, which exhibit a strikingly low level of gene methylation.^14,15^

As found in other classes of cancer, the hypermethylation we see in the YST as compared with seminoma cells is restricted to the CpG islands of a relatively small proportion of genes, a so-called CpG island methylator phenotype. Analysis of EC and teratoma cells revealed a much less localised difference in methylation. In these cells, the extent to which methylation of CpGs was higher than in seminoma cells was almost uniform across CpGs in all regions. This hypermethylation does not, therefore, represent a CpG island methylator phenotype. This implies a much less biologically regulated process than the targeted methylation of CpG islands seen in YST cells. However, despite the fact that the methylation did not appear to be targeted to CpG islands in EC/teratoma cells, there was still a strong inverse correlation between CpG island methylation and the level of gene expression. Hence, regardless of the mechanism that results in CpG island hypermethylation, such methylation shows a strong correlation with gene silencing.

### CpG island methylation shows the strongest correlation with reduced gene expression

Methylation of CpGs islands was more strongly correlated with low gene expression than was methylation in other regions. With respect to gene structure itself, most methylated islands were in the region of gene promoters. However, some were in the gene body where they similarly correlated with gene silencing. Methylation of CpG islands in gene bodies has been reported to be associated with active genes.^23,24^ Therefore, the situation in GCT cell lines reveals a distinctly different relationship. Recent studies have implicated a variety of differentially methylated regions as most influential in the regulation of gene expression. Kulis *et al.*^25^ found that in differentiated B-cells and leukaemic cells differences in methylation in the gene bodies and promoter-associated CpGs correlated with differences in gene expression, but the correlation was stronger for gene bodies.^25^ In their study, as has been found before, increased methylation in gene bodies correlated more often with increased expression rather than decreased expression, although numerous genes did exhibit the latter. Irizarry *et al.*^26^ found that differences in methylation of CpG shores correlated best with differences in gene expression in both tissue-specific and cancer-specific differentially methylated regions.^26^ More recently a whole genome bisulfite sequencing study in medulloblastomas identified regions ~2 kb downstream of the transcription start site as showing the strongest correlation with gene expression.^27^ Hence, it seems that the role of methylation of different gene regions in controlling gene expression varies in different tissue types and different cancers. It will be interesting in the future to find whether the situation seen in GCT cell lines is an unusual feature of their germ cell lineage, or a more general phenomenon in a range of cancers.

### DNA methylation and cancer progression in GCTs

Our finding that the non-seminoma cell lines exhibit very high levels of gene methylation as compared with seminoma cells is consistent with earlier, less comprehensive studies, which showed that non-seminomas exhibit much higher levels of gene methylation than seminomas.^14,15^

In previous studies, using immunostaining, DNA methylation was undetectable in ICGNU and most seminomas while strong methylation was seen in non-seminomas.^16,28^ Even in mixed tumours, seminoma elements were unmethylated and non-seminoma components methylated.^16^ This led Netto *et al.*^16^ to propose that ICGNUs are derived from PGCs at an embryonic stage when methylation has been erased, and seminomas subsequently arise from ICGNU. Given that the seminoma cell line, TCAM2, can give rise to tumours closely resembling EC in an experimental *in vivo* context^29^ and tumours containing a mixture of seminomatous and non-seminomatous components are not uncommon, it seems possible that hypermethylation could represent a progression event involving *de novo* methylation of such seminomatous components. It therefore seems plausible that pure non-seminomas could also arise by *de novo* methylation from a non-methylated ICGNU or seminoma precursor lesion that existed prior to diagnosis. This would be consistent with the well-documented phenomenon of non-seminomas arising as recurrences of seminomas.^13^

### Cell lines as a useful system for methylation studies

As shown here, the correlation between substantial methylation of CpG islands and gene silencing is well below 100%. It is therefore critical that DNA methylation is not simply assumed to be the cause of gene silencing. In this study, in addition to determining the level of differential methylation that best correlated with gene silencing, we were able to utilise these cell lines to verify the role of methylation using the DNA-demethylating agent 5-aza-2-deoxycytidine. To determine whether silencing of these genes was a more general phenomenon in primary tumours, we showed that there was a highly significant overlap in the genes silenced by methylation in the cell lines in this study and those genes that differed in their expression in a cohort of paediatric GCT samples. Although this does not determine if differences in expression in the tumour samples are due to their methylation, it is the difference in expression that is the biologically important outcome of the methylation. Analysis of primary tumour samples using lower coverage analyses has already shown the same general differences in the level of methylation between seminoma and non-seminoma tumours that we found in the corresponding cell lines,^14,15^ supporting the hypothesis that the methylation seen in the cell lines is a reflection of that seen in primary tumours.

Concerns have been raised over using cell lines for methylation studies.^30^ Smiraglia *et al.*^30^ concluded that cancer cell lines develop aberrant methylation profiles as an artifact of the culture process.^30^ Although this possibility cannot be ruled out, this is not, in our view, the only or indeed the most likely interpretation of the data in their study. Smiraglia *et al.*^30^ showed, using restriction landmark genomic scanning, that the high level of methylation seen in some cancer cell lines was not reflected in primary tumours of the same type (although not the actual tumour samples from which those cell lines were derived). Their conclusion is based on the assumption that the average methylation state of all the cells in most of a given class of tumour should be similar to that found in a cell line derived from that tumour. However, an alternative explanation for their observations, for which there is clear precedent, is that the cell line methylation status reflects the minority population of tumour cells from which the cell lines originate. These cells are a proliferating subset of cells within the tumours, which could represent the cancer stem cell/tumour initiating cell. Such a difference in DNA methylation clearly exists in normal tissues of a single individual *in vivo* that, like tumours, are genetically clonal. This is true of different cell populations in the same tissue^31,32^ and differentiated cells versus proliferating stem cells in whole organisms.^33^ In such examples the gene methylation profiles of genetically identical cells can be dramatically different *in vivo*, just as the profile of a cancer stem cell or cancer-derived cell line might differ from less primitive proliferating cells or their more differentiated progeny in the primary tumour.

Indeed, using a more sensitive analysis of cell lines and the pancreatic cancers from which they were derived, Ueki *et al.*^34^ (using methylation-specific PCR) concluded that ‘most of the DNA methylation of tumour suppressor genes observed in cancer cell lines is present in the primary carcinoma from which they were derived.^34^

Given our observations that the seminoma cell line exhibits no apparent increase in methylation, that the hypermethylation we identified in one EC cell line was very similar to that seen in a different cell line in another study van der Zwan *et al.*^18^ and that the overall pattern of methylation we found is consistent with earlier studies of primary tumour samples, it seems likely that the majority of methylation events we see reflect genuine differences between these tumour types.

### Several pluripotency regulators are silenced in non-seminoma cell lines

Of the 108 genes silenced by methylation in the YST cell line, 21 were also similarly differentially expressed in primary tumour samples (Table 2). Among these 21 genes several have previously been associated with germ cell progenitors and/or pluripotency.

KLF4 is one of the four ‘Yamanaka’ factors that together can endow somatic cells with the potential to adopt a pluripotent embryonic stem cell-like state^35^ and is highly expressed in the pluripotent progenitors of germ cells, the PGCs.^36,37^ Its suppression by methylation in non-seminomas might therefore play a key role in their more differentiated and more aggressive state. It is also noteworthy that *Klf4* expression is affected by the protein encoded by another silenced gene, *Prdm14*^38^ (discussed below) and that silencing Tet1 in mice (which results in hypermethylation of DNA) downregulates the expression of *Klf4* and *Prdm14*.^39^ Therefore, silencing of these two genes may be intrinsically linked.

Among the other genes identified, several have particular roles during male gamete production. DDX43 and TDRD12 were specifically hypomethylated in seminoma cells. DDX43 encodes a ‘cancer testis antigen’ (also called HAGE), which is an RNA-dependent helicase with expression largely restricted to testis and a variety of cancer types.^40^ TDRD12 encodes a tudor domain-containing protein (also capable of functioning as an RNA-dependent helicase) found almost exclusively in testes.^41^ It is important in the biogenesis of piRNAs, which are also testis specific.^41^ Hence the heavy methylation of these genes in all control samples is consistent with their very restricted expression patterns in normal tissues. This implies that the difference in expression of these two genes between GCT subtypes is due to hypomethylation in seminoma (and teratoma for TDRD12). MNS1 and RBMXL2 on the other hand were hypermethylated in YST cells. *MNS1* (meiosis-specific nuclear structural 1) encodes a coiled-coil protein of unknown function, which is essential for spermiogenesis.^42^ *RBMXL2* (also called hnRNP G-T) codes for an hnRNP expressed almost exclusively in testes and GCTs believed to function during meiotic prophase or to act as a germ cell-specific splicing regulator.^43^ Hence, the silencing of these genes in non-seminomas may also play a role in their differentiation towards somatic cell lineages.

### PRDM14

PRDM14, which is differentially methylated and expressed between the seminoma and YST cell lines (methylated and silenced in YST), merits special attention. It encodes a multiple zinc finger transcription factor almost exclusively expressed in PGCs, GCTs and blastocyst stage embryos (NCBI EST profile http://www.ncbi.nlm.nih.gov/IniGene/ESTProfileViewer.cgi?uglist=Hs.287532), which can function as both an activator and repressor of transcription and may possess DNA methyltransferase activity. Alongside PRDM1 (BLIMP1), PRDM14 activates *TFAP2c* (which encodes AP2γ) and together these three ‘key regulators of PGC specification’^44–46^ repress somatic gene expression, activate PGC genes and initiate the demethylation of the genome. Together these factors can convert ES cells to PGCs.^44–46^ PRDM14 actively promotes DNA demethylation by directly repressing the DNA methytransferases, *DNMT3a*, *DNMT3b*, *DNMT3l* and *UHRF1* (a DNMT1 cofactor)^38,47,48^ and directly activating DNA demethylation by increasing the activity of the TET enzymes.^48^

In mice, Prdm14 can also enhance reprogramming of somatic cells to iPS cells by Sox2, Oct4 and Klf4.^49^ It regulates Oct4^49^ and can even induce pluripotency when overexpressed alongside Blimp1 and Prmt5.^50^

Increased copy number of the *PRDM14* gene has been reported in GCTs^51^ and a recent GWAS study identified *PRDM14* as a susceptibility locus in testicular cancer.^52^ Studies in normal ES cells (where *PRDM14* is also expressed but at much lower levels than in PGCs) provide evidence for a role in the progression of GCTs towards the YST phenotype. In one study, knockdown of PRDM14 led to differentiation towards extraembryonic endoderm,^47^ a tissue type similar to that seen in YSTs. However, this remains somewhat contentious since, in a similar study by others, knockdown of PRDM14 caused cells to differentiate into embryonic cell types.^53^

Together, these data suggest that PRDM14 could play a central role in GCT progression in which it is initially expressed in seminomas hence helping to retain their germ cell-like phenotype. It is then silenced by methylation triggering more widespread methylation of the genome and promoting the cells’ differentiation into the extraembryonic cell types that typify YSTs.

### Conclusions

This study revealed a very different methylator phenotype in non-seminoma cell lines as compared with other types of cancer. Several new potentially biologically important genes were identified, most particularly a group of genes associated with the germ cell state and/or pluripotency—*PRDM14*, *TDRD12*, *DDX43*, *MNS1*, *RBMXL2* and *Klf4*. Silencing of these factors that normally suppress somatic differentiation could be a mandatory step in the progression from seminoma to non-seminoma. Both the silenced genes and gene methylation generally represent new potential therapeutic targets for the more chemoresistant GCTs.

## Materials and methods

### Cell culture and 5-deoxyazacytidine treatment

Adult GCT cell lines were TCAM2 (seminoma^54,55^), GCT27 (EC^56^), NT2D1 (teratoma^56^) and GCT44 (YST^57^).

These were cultured in Dulbecco’s Modified Eagle Medium (EC, teratoma and YST) and RPMI-1640 media (seminoma) (Sigma-Aldrich, Dorset, UK) containing 10% foetal bovine serum (Sigma-Aldrich) and 1% penicillin/streptomycin (Sigma-Aldrich). For demethylation experiments 1×10^5^ YST cells were treated with 5 μM 5-deoxyazacytidine (Sigma). After 24 h, medium was replaced with a fresh medium without 5-deoxyazacytidine then cells were left for one further day. Cells were then trypsinized for expression analysis.

### Methylation analysis

Bisulfite conversion of the DNA (1 μg) was performed using EZ DNA methylation kit (Zymo Research Corporation, Irvine, CA, USA), with hybridisation to the Infinium HumanMethylome450 arrays (Illumina, San Diego, CA, USA) and scanning performed by the Queen Mary University of London Genome Centre. Quality control of the dataset was performed by analysing the bisulfite conversion using Genome Studio software (Illumina). The ratio of unmethylated probe to methylated probe was calculated with samples all showing good conversion rations <0.2. Low signal intensity was also controlled for by removing CpG probes with an averaged detection *P* value of >0.05 across samples. In addition, averaged signal intensity values for each CpG, for both the red and green signals, across the samples were log2 transformed with values <11.1 removed. For the analysis shown here, probes that bound multiple sites (chr-MULTI) were excluded.

CpGs were annotated by the chip manufacturers according their position relative to CpG islands: 2,000 bp either side of an island as north ‘shore’ (5′ relative to the associated gene) or south shore (3’ relative to the associated gene). North and south ‘shelves’ 2,000 bp flanking the shores, and ‘other’ or ‘open sea’ for CpGs more distant from an island.’ Quantitative measurements of DNA methylation across all known genes and CpGs were represented as *β*-values (0<*β*<1, 0 represents unmethylated sites, and 1 indicates the site is fully methylated). Δ*β*-values of differential methylation were calculated as the difference between the *β*-values of each cell line relative to the seminoma cell line. The Excel tool PivotTable (Microsoft office 2010) was used to assign average methylation and differential methylation measurements to each gene according to various combinations of locations with respect to CpG islands and gene regions. Probes labelled by multiple gene names were excluded from the analysis.

For comparison of normal tissue against subset of CpG probes, Marmal-aid software was utilised^58^ with clustering using R program ‘fast cluster’ (www.bioconductor.org). Normal tissue included 4 prostate samples (GEO accession GSE38240—sample identifiers GSM937263, GSM937265, GSM937267 and GSM937269), 5 blood samples (GSE41169—sample identifiers GSM1009666, GSM1009667, GSM1009668, GSM1009686 and GSM1009688) and 12 blood samples from Heyn *et al.*, ^59^ (4 new born—CB15, CB76, CB23 and CB9; 5 adult—MA1, MA16, A29, A30 and A31 and 3 adults >80 years old—OLD16, OLD17 and OLD18). Hierarchical clustering of probes within CpG islands were performed using ward’s method, with *β*-values represented as heatmaps.

### Expression array analysis

RNA extraction was performed using the RNeasy extraction kit (Qiagen, Manchester, UK) according to the manufacturer's protocol. RNA was eluted with RNAse-free water (Qiagen). The quality of TGCT RNA samples was determined using a Bioanalyzer 2100 (Agilent Technologies, Santa Clara, CA, USA) as suggested by the manufacturer. Measurement was calculated using 2100 expert software version B.02.07 (Agilent Technologies) and displayed as RNA concentration, the ribosomal ratio and the RNA integrity number (RIN). For the purpose of selecting samples for Affymetrix Gene Expression array, samples with RIN>9.0 were selected as this implies a high-quality RNA sample. Arrays were performed at the Nottingham Arabidopsis Stock Centre, University of Nottingham Sutton Bonington Campus using Affymetrix GeneChip Human Genome U133 Plus 2.0 arrays (the University of Nottingham).

Data were first preprocessed using the statistical software, R with ‘Affy’ package provided by www.bioconductor.org. Data were normalised using the RMA method^60^ and filtered such that probes which gave expression outputs below control background probes (recorded in the GeneChip) for all four cell lines were excluded. Fold changes in expression between each probe of each cell lines relative to Seminoma were calculated, and annotation packages were used to assign gene information to each probe set. The data were exported as a.txt file to be read and analysed in Excel. The Excel tool PivotTable was used to assign average expression intensity values to each gene.

CEL files from the studies by Palmer *et al.*^22^ and van der Zwan *et al.*^18^ were processed using the statistical package, R. Data were normalised using the RMA method, and filtered to exclude outputs that fell below background levels. These data were then processed in Excel and a one-tailed (right tail) Welch’s *t*-test was performed for each gene comparing Seminoma samples with non-seminoma samples (*P* value of <0.05 indicated significantly higher expression in seminoma samples versus YST samples).

### RT–PCR and RT–qPCR

RNA was extracted in TRI Reagent (Sigma) and treated with DNAse (Invitrogen, Paisley, UK). Complementary DNA was synthesised using random primer mix (Promega, Southampton, UK) and 200 ng of Oligo(dT)18 Primer (Fermentase Hanover, NH, USA) and reverse transcriptase (Invitrogen). PCR was carried out using Phusion High-Fidelity PCR Master Mix (NEB, Herts, UK).

For RT–qPCR, a master mix for each primer pair was prepared by mixing 2X Brilliant SYBR Green QPCR Master Mix (Agilent), 100 nM of forward and reverse primer, and water up to 25 ml/well. About 25 μl of master mix was transferred into each designated well of a 96-well plate before adding 50 ng complementary DNA into each well. Samples were then subjected to PCR using Bio-Rad C1000 Thermal Cycler machine (Bio Rad, Heatfords, UK). The PCR cycling condition are as follows: 95 °C for 3 min followed by 35 cycles of 95 °C for 1 min, 58 °C for 1 min and 72 °C for 30 s then 72 °C for 5 min. All data were then analysed using Bio-Rad CFX Manager 3.0 software (Bio Rad).

The threshold cycle (Ct) values of each sample was defined by standard threshold and the relative comparison with the housekeeping gene, ACTB, was calculated using the Pfaffl equation.^61^

No ethical approval was required for this study.

## DATA DEPOSIT

The array data presented in this paper have been deposited in the GEO database.

## Figures and Tables

**Table 1:** Contingency tables of observed and expected number of genes differentially expressed (correlating and anti-correlating), and genes with no difference in expression for ranges of differential methylation between non-seminoma and seminoma cell lines

*Δ*β	*SEM/YST*		*No difference* *Observed*	*No difference* *Expected*	*YST/SEM*		*Total*	P *value*	*Significance*
	*Genes >2-fold*	*Genes >2-fold*			*Genes >2-fold*	*Genes >2-fold*		Χ^2^ *(2df)*	
	*Observed*	*Expected*			*Observed*	*Expected*			
0.9–0.95	2	0.75	2	2.86	0	0.39	4	0.2537	
0.85–0.9	3	1.31	4	5.01	0	0.68	7	0.2157	
0.8–0.85	16	5.42	10	20.75	3	2.82	29	0	***
0.75–0.8	16	4.86	8	18.6	2	2.53	26	0	***
0.7–0.75	16	5.61	9	21.47	5	2.92	30	0	***
0.65–0.7	11	5.42	16	20.75	2	2.82	29	2.93E−02	*
0.6–0.65	12	6.55	21	25.04	2	3.41	35	5.57E−02	
0.55–0.6	7	5.05	17	19.32	3	2.63	27	0.5819	
0.5–0.55	13	7.67	26	29.34	2	3.99	41	0.0789	
0.45–0.5	15	9.17	30	35.06	4	4.77	49	0.1018	
0.4–0.45	21	10.66	29	40.79	7	5.55	57	0.001	***
0.35–0.4	16	13.66	49	52.23	8	7.11	73	0.6998	
0.3–0.35	29	16.46	47	62.97	12	8.57	88	0.0006	***
0.25–0.3	29	20.02	64	76.56	14	10.42	107	0.0257	*
0.2–0.25	40	28.43	90	108.76	22	14.81	152	0.0033	**
0.15–0.2	34	34.98	128	133.8	25	18.22	187	0.2458	
0.1–0.15	57	50.88	185	194.62	30	26.49	272	0.4326	
0.05–0.1	96	78.57	279	300.52	45	40.91	420	0.0545	
0–0.05	339	466.53	1,939	1,784.54	216	242.93	2,494	0.00E+00	***
Total	772	772	2,953	2,953	402	402	4,127		

*Δ*β	*SEM/TERT*		*No difference* *Observed*	*No difference* *Expected*	*TERT/SEM*		*Total*	P *value*	*Significance*
	*Genes >2-fold*	*Genes >2-fold*			*Genes >2-fold*	*Genes >2-fold*		Χ^2^ *(2df)*	
	*Observed*	*Expected*			*Observed*	*Expected*			
0.9–0.95	0	0	0	0	0	0	0	0	
0.85–0.9	2	0.72	2	2.83	0	0.45	4	0.2225	
0.8–0.85	6	2.5	8	9.9	0	1.58	14	0.0327	*
0.75–0.8	7	1.61	2	6.37	0	1.02	9	0.00001	***
0.7–0.75	4	1.07	2	4.25	0	0.68	6	0.0072	**
0.65–0.7	6	3.04	9	12.03	2	1.93	17	1.61E−01	
0.6–0.65	6	3.39	11	13.45	2	2.16	19	2.94E−01	
0.55–0.6	3	3.22	12	12.74	3	2.04	18	0.776	
0.5–0.55	6	4.11	14	16.28	3	2.61	23	0.537	
0.45–0.5	6	4.47	17	17.69	2	2.84	25	0.6713	
0.4–0.45	5	4.65	19	18.4	2	2.95	26	0.8387	
0.35–0.4	8	4.83	14	19.11	5	3.06	27	0.0965	
0.3–0.35	9	6.08	18	24.06	7	3.86	34	0.0642	
0.25–0.3	9	10.55	40	41.76	10	6.69	59	0.38001	
0.2–0.25	19	17.17	58	67.94	19	10.89	96	0.0214	*
0.15–0.2	30	24.49	90	96.96	17	15.54	137	0.3921	
0.1–0.15	33	42.38	172	167.73	32	26.89	237	0.2064	
0.05–0.1	72	71.7	279	283.8	50	45.49	401	0.7676	
0–0.05	245	270.01	1,117	1,068.69	148	171.31	1510	0.0216	*
Total	476	476	1,884	1,884	302	302	2,662		

*Δ*β	*SEM/EC*		*No difference* *Observed*	*No difference* *Expected*	*EC/SEM*		*Total*	P *value*	*Significance*
	*Genes >2-fold*	*Genes >2-fold*			*Genes >2-fold*	*Genes >2-fold*		Χ^2^ *(2df)*	
	*Observed*	*Expected*			*Observed*	*Expected*			
0.9–0.95	0	0	0	0	0	0	0	0	
0.85–0.9	2	0.29	0	1.5	0	0.19	2	0.0031	**
0.8–0.85	10	2.51	7	12.79	0	1.69	17	1.60E−06	***
0.75–0.8	11	2.66	7	13.54	0	1.79	18	1.76E−07	***
0.7–0.75	11	3.25	10	16.55	1	2.19	22	0.00002	***
0.65–0.7	7	4.14	17	21.07	4	2.79	28	0.1935	
0.6–0.65	5	3.55	17	18.06	2	2.39	24	6.97E−01	
0.55–0.6	6	4.73	23	24.08	3	3.19	32	0.8183	
0.5–0.55	11	6.06	29	30.85	1	4.09	41	0.0393	*
0.45–0.5	6	4.88	20	24.83	7	3.29	33	0.0682	
0.4–0.45	7	5.32	26	27.09	3	3.59	36	0.7149	
0.35–0.4	5	6.06	33	30.85	3	4.09	41	0.7312	
0.3–0.35	8	9.9	51	50.41	8	6.68	67	0.729	
0.25–0.3	11	13.15	68	66.97	10	8.88	89	0.7749	
0.2–0.25	21	16.7	74	85.02	18	11.27	113	0.0378	*
0.15–0.2	20	18.03	85	91.79	17	12.17	122	0.2681	
0.1–0.15	32	32.96	169	167.79	22	22.25	223	0.9805	
0.05–0.1	56	59.86	298	304.73	51	40.41	405	0.2045	
0–0.05	288	322.94	1,698	1,644.06	199	218	2,185	0.0272	*
Total	517	517	2,632	2,632	349	349	3,498		

Abbreviations: EC, embryonal carcinoma; df, degree of freedom; SEM, seminoma; TERT, teratoma; YST, yolk sac tumour. *P* values of the *Χ*^2^-test of association between methylation and expression are given. *P<0.05

**Table 2:** List of the genes identified as differentially methylated and differentially expressed between cell lines that were also differentially expressed in the same way in primary seminoma and non-seminoma tumour samples^22^

*A. Seminoma versus YST*			
*Gene*	*Noor (2015)*	*Korkola* et al.^21^	*Palmer* et al.^22^
*EPCAM*	**561.2**	0.4	0.4
*TACSTD2*	**448.8**	0.1	0.9
*GGCT*	**120.4**	1.0	1.5
*F2RL1*	**106.1**	0.9	0.8
*TDRD12*	**84.4**	**8.5**	**8.0**
*FABP5*	**65.7**	1.4	0.5
*HSPA2*	**50.7**	0.9	0.9
*SOX17*	**48.7**	0.8	0.6
*RBMXL2*	**43.5**	**2.3**	1.4
*BASP1*	**42.0**	0.8	1.3
*DDX43*	**37.3**	**3.6**	**3.9**
*KLF4*	**29.9**	**5.9**	**7.2**
*LGALS3*	**28.1**	0.8	**1.6**
*ESRP2*	**27.7**	0.3	0.5
*CYB5R2*	**18.2**	1.1	1.0
*OXCT1*	**17.2**	1.2	**1.9**
*HIST1H4C*	**17.1**	**1.5**	0.9
*CLDN7*	**15.2**	0.4	0.2
*ALDH1A3*	**14.6**	1.4	0.4
*ECHDC3*	**14.5**	0.9	**1.8**
*TRIL*	**14.4**	**1.9**	1.4
*TMEM168*	**14.2**	1.4	**2.2**
*CXCL14*	**13.0**	**2.7**	**5.1**
*DSCR6*	**10.2**	0.5	0.2
*GPX7*	**9.6**	1.0	1.0
*FAM184A*	**9.4**	0.4	0.5
*ECHDC2*	**9.2**	0.5	0.9
*IER2*	**9.0**	1.0	**1.5**
*GUCA1A*	**7.5**	**1.8**	1.2
*LGALS8*	**7.4**	0.6	0.9
*ST14*	**6.7**	0.7	0.5
*MAPK13*	**6.3**	0.5	0.8
*LMO7*	**6.2**	0.6	0.8
*OVOL1*	**6.2**	0.8	0.8
*LY75*	**6.0**	**3.5**	**3.4**
*CYB5R1*	**5.6**	1.0	1.3
*GIPC2*	**5.6**	0.6	0.8
*EPS8L2*	**5.5**	0.4	0.4
*CST6*	**4.8**	0.8	1.2
*PKP3*	**4.7**	0.4	0.3
*ALX1*	**4.6**	1.0	0.8
*PARP12*	**4.3**	**3.9**	**2.6**
*BST1*	**4.3**	**1.5**	1.5
*CYP1B1*	**4.3**	1.0	**1.8**
*MNS1*	**3.6**	1.0	1.0
*SOX15*	**3.5**	**5.9**	**3.3**
*MTL5*	**3.5**	0.8	0.8
*MYD88*	**3.3**	1.0	0.9
*PRDM14*	**3.2**	**1.6**	1.2
*MEIS1*	**3.2**	0.5	0.4
*TNFRSF10C*	**3.1**	0.9	1.0
*TRIM25*	**3.1**	1.0	0.9
*PON3*	**3.0**	**1.6**	**4.9**
*PLXND1*	**3.0**	1.0	0.9
*RPRM*	**3.0**	**2.3**	**2.4**
*LTBP2*	**2.9**	1.1	0.6
*FUT1*	**2.8**	1.1	1.0
*SLC25A38*	**2.8**	0.8	0.8
*EPS8L1*	**2.6**	0.8	0.7
*ARPC1B*	**2.6**	**2.3**	**2.9**
*EDNRB*	**2.6**	0.9	0.9
*TEAD3*	**2.5**	0.6	0.7
*ACADL*	**2.5**	1.1	0.9
*TUBB6*	**2.5**	0.9	0.6
*ZMIZ1*	**2.4**	**1.6**	1.1
*ABHD14A*	**2.2**	0.8	0.9
*ELMO3*	**2.2**	0.8	0.7
*AMPD3*	**2.1**	1.1	1.3
*LRRFIP1*	**2.0**	1.3	1.2
*ZIC1*	**2.0**	0.5	0.7
*SH2D3A*	**1.6**	1.0	1.0
*NAAA*	**1.5**	1.1	1.1

*B. Seminoma versus EC*		
*Gene*	*Noor (2015)*	*Korkola* et al.^21^
*TACSTD2*	**322.3**	0.3
*NUDT15*	**96.1**	0.6
*RBMXL2*	**52.8**	**1.6**
*HSPA2*	**35.6**	0.6
*DDX43*	**32.4**	**2.1**
*TDRD12*	**27.3**	**5.3**
*UCP2*	**24.6**	0.8
*MGLL*	**18.4**	0.6
*CRIP1*	**15.5**	1.1
*RPL39L*	**13.4**	1.4
*CHODL*	**12.1**	1.2
*MGMT*	**10.4**	1.2
*CYB5R2*	**8.5**	0.9
*CYP2R1*	**8.0**	**1.6**
*KRT7*	**7.6**	0.6
*PRKCH*	**6.0**	0.6
*MAPK13*	**6.0**	0.8
*CNTLN*	**5.8**	1.2
*CXCR7*	**5.7**	0.4
*CTSO*	**5.0**	0.7
*ST14*	**5.0**	0.8
*BST1*	**4.6**	1.3
*GREM2*	**4.5**	0.9
*DMRT1*	**4.4**	**5.1**
*SLC7A4*	**3.9**	1.3
*LTBP2*	**2.8**	0.4
*KDELR3*	**2.6**	0.6
*PON3*	**2.5**	**2.3**

*C. Seminoma versus teratoma*		
*Gene*	*Noor (2015)*	*Korkola* et al.^21^
*TACSTD2*	**438.9**	0.0
*DDX43*	**43.7**	**4.5**
*RBMXL2*	**42.8**	**2.2**
*ME1*	**35.4**	0.5
*RBM12B*	**21.9**	1.2
*QPCT*	**20.4**	0.6
*PRKCH*	**14.7**	0.7
*CAT*	**12.9**	0.4
*GALNT3*	**12.7**	0.2
*ECHDC2*	**8.9**	0.6
*PKP3*	**6.7**	0.6
*ST14*	**6.7**	0.5
*GREM2*	**4.9**	0.5
*SOX15*	**3.8**	**5.3**
*EDNRB*	**3.1**	0.4
*PON3*	**2.9**	**1.6**
*CYP1B1*	**2.5**	0.3
*EPS8L1*	**2.5**	0.8
*SULT1A1*	**2.5**	1.0
*LPHN3*	**2.3**	0.7
*VAV1*	**2.3**	1.1
*TEAD3*	**2.2**	0.7
*ELMO3*	**2.0**	0.8

Abbreviations: EC, embryonal carcinoma; YST, yolk sac tumour.

The ‘fold change’ column is the fold change in expression seen between the seminoma and non-seminoma cell lines in our study. The ‘Korkola fold change’ and ‘Palmer fold change’ are the difference in expression seen in primary tumours in the studies by Korkola *et al.*^21^ and Palmer *et al.*^22^ Only genes for which data were available in all data sets are shown. Genes where expression in seminoma cells is at least 1.5-fold greater than in the non-seminoma cells are in bold.

## References

[bib1] Feinberg, A. P. & Vogelstein, B. Hypomethylation distinguishes genes of some human cancers from their normal counterparts. Nature 301, 89–92 (1983). 6185846 10.1038/301089a0

[bib2] Feinberg, A. P. & Tycko, B. The history of cancer epigenetics. Nat. Rev. Cancer 4, 143–153 (2004). 14732866 10.1038/nrc1279

[bib3] Teodoridis, J. M. , Hardie, C. & Brown, R. CpG island methylator phenotype (CIMP) in cancer: causes and implications. Cancer Lett. 268, 177–186 (2008). 18471961 10.1016/j.canlet.2008.03.022

[bib4] Toyota, M. et al. CpG island methylator phenotype in colorectal cancer. *Proc. Natl Acad. Sci. USA* 96, 8681–8686 (1999). 10411935 10.1073/pnas.96.15.8681 PMC17576

[bib5] Weisenberger, D. J. et al. CpG island methylator phenotype underlies sporadic microsatellite instability and is tightly associated with BRAF mutation in colorectal cancer. Nat. Genet. 38, 787–793 (2006). 16804544 10.1038/ng1834

[bib6] Shen, L. et al. Integrated genetic and epigenetic analysis identifies three different subclasses of colon cancer. *Proc. Natl Acad. Sci. USA* 104, 18654–18659 (2007). 18003927 10.1073/pnas.0704652104 PMC2141832

[bib7] Noushmehr, H. et al. Identification of a CpG island methylator phenotype that defines a distinct subgroup of glioma. Cancer Cell 17, 510–522 (2010). 20399149 10.1016/j.ccr.2010.03.017 PMC2872684

[bib8] Motzer, R. J. et al. Testicular cancer. J. Natl. Compr. Canc. Netw. 10, 502–535 (2012). 22491049 10.6004/jnccn.2012.0050

[bib9] Beyer, J. et al. Maintaining success, reducing treatment burden, focusing on survivorship: highlights from the third European consensus conference on diagnosis and treatment of germ-cell cancer. Ann Oncol. 24, 878–888 (2013). 23152360 10.1093/annonc/mds579 PMC3603440

[bib10] Matsutani, M. et al. Primary intracranial germ cell tumors: a clinical analysis of 153 histologically verified cases. J. Neurosurg. 86, 446–455 (1997). 9046301 10.3171/jns.1997.86.3.0446

[bib11] Murray, M. J. et al. The two most common histological subtypes of malignant germ cell tumour are distinguished by global microRNA profiles, associated with differential transcription factor expression. Mol. Cancer 9, 290 (2010). 21059207 10.1186/1476-4598-9-290 PMC2993676

[bib12] Looijenga, L. H. Human testicular (non)seminomatous germ cell tumours: the clinical implications of recent pathobiological insights. J. Pathol. 218, 146–162 (2009). 19253916 10.1002/path.2522

[bib13] Tan, C. & Scotting, P. J. Stem cell research points the way to the cell of origin for intracranial germ cell tumours. J. Pathol. 229, 4–11 (2013). 22926997 10.1002/path.4098

[bib14] Jeyapalan, J. N. et al. Methylator phenotype of malignant germ cell tumours in children identifies strong candidates for chemotherapy resistance. Br. J. Cancer 105, 575–585 (2011). 21712824 10.1038/bjc.2011.218 PMC3170957

[bib15] Amatruda, J. F. et al. DNA methylation analysis reveals distinct methylation signatures in pediatric germ cell tumors. BMC Cancer 13, 313 (2013). 23806198 10.1186/1471-2407-13-313 PMC3701494

[bib16] Netto, G. J. et al. Global DNA hypomethylation in intratubular germ cell neoplasia and seminoma, but not in nonseminomatous male germ cell tumors. Mod. Pathol. 21, 1337–1344 (2008). 18622385 10.1038/modpathol.2008.127 PMC4086525

[bib17] Rijlaarsdam, M. A. et al. Genome wide DNA methylation profiles provide clues to the origin and pathogenesis of germ cell tumors. PLoS ONE 10, e0122146 (2015). 25859847 10.1371/journal.pone.0122146 PMC4479500

[bib18] van der Zwan, Y. G. et al. Seminoma and embryonal carcinoma footprints identified by analysis of integrated genome-wide epigenetic and expression profiles of germ cell cancer cell lines. PLoS ONE 9, e98330 (2014). 24887064 10.1371/journal.pone.0098330 PMC4041891

[bib19] Estecio, M. R. & Issa, J. P. Dissecting DNA hypermethylation in cancer. FEBS Lett. 585, 2078–2086 (2011). 21146531 10.1016/j.febslet.2010.12.001 PMC3378045

[bib20] Sadakierska-Chudy, A. , Kostrzewa, R. M. & Filip, M. A comprehensive view of the epigenetic landscape part I: DNA methylation, passive and active DNA demethylation pathways and histone variants. Neurotox. Res. 27, 84–97 (2015). 25362550 10.1007/s12640-014-9497-5 PMC4286137

[bib21] Korkola, J. E. et al. Down-regulation of stem cell genes, including those in a 200-kb gene cluster at 12p13.31, is associated with in vivo differentiation of human male germ cell tumors. Cancer Res. 66, 820–827 (2006). 16424014 10.1158/0008-5472.CAN-05-2445

[bib22] Palmer, R. D. et al. Pediatric malignant germ cell tumors show characteristic transcriptome profiles. Cancer Res. 68, 4239–4247 (2008). 18519683 10.1158/0008-5472.CAN-07-5560

[bib23] Stirzaker, C. , Taberlay, P. C. , Statham, A. L. & Clark, S. J. Mining cancer methylomes: prospects and challenges. Trends Genet. 30, 75–84 (2014). 24368016 10.1016/j.tig.2013.11.004

[bib24] Aran, D. , Sabato, S. & Hellman, A. DNA methylation of distal regulatory sites characterizes dysregulation of cancer genes. Genome Biol. 14, R21 (2013). 23497655 10.1186/gb-2013-14-3-r21 PMC4053839

[bib25] Kulis, M. et al. Epigenomic analysis detects widespread gene-body DNA hypomethylation in chronic lymphocytic leukemia. Nat. Genet. 44, 1236–1242 (2012). 23064414 10.1038/ng.2443

[bib26] Irizarry, R. A. et al. The human colon cancer methylome shows similar hypo- and hypermethylation at conserved tissue-specific CpG island shores. Nat. Genet. 41, 178–186 (2009). 19151715 10.1038/ng.298 PMC2729128

[bib27] Hovestadt, V. et al. Decoding the regulatory landscape of medulloblastoma using DNA methylation sequencing. Nature 510, 537–541 (2014). 24847876 10.1038/nature13268

[bib28] Wermann, H. et al. Global DNA methylation in fetal human germ cells and germ cell tumours: association with differentiation and cisplatin resistance. J. Pathol. 221, 433–442 (2010). 20593487 10.1002/path.2725

[bib29] Nettersheim, D. et al. Establishment of a versatile seminoma model indicates cellular plasticity of germ cell tumor cells. Genes Chromosomes Cancer 51, 717–726 (2012). 22489004 10.1002/gcc.21958

[bib30] Smiraglia, D. J. et al. Excessive CpG island hypermethylation in cancer cell lines versus primary human malignancies. Hum. Mol. Genet. 10, 1413–1419 (2001). 11440994 10.1093/hmg/10.13.1413

[bib31] Lister, R. et al. Global epigenomic reconfiguration during mammalian brain development. Science 341, 1237905 (2013). 23828890 10.1126/science.1237905 PMC3785061

[bib32] Bocker, M. T. et al. Genome-wide promoter DNA methylation dynamics of human hematopoietic progenitor cells during differentiation and aging. Blood 117, e182–e189 (2011). 21427290 10.1182/blood-2011-01-331926

[bib33] Meissner, A. et al. Genome-scale DNA methylation maps of pluripotent and differentiated cells. Nature 454, 766–770 (2008). 18600261 10.1038/nature07107 PMC2896277

[bib34] Ueki, T. et al. Aberrant CpG island methylation in cancer cell lines arises in the primary cancers from which they were derived. Oncogene 21, 2114–2117 (2002). 11960385 10.1038/sj.onc.1205275

[bib35] Takahashi, K. & Yamanaka, S. Induction of pluripotent stem cells from mouse embryonic and adult fibroblast cultures by defined factors. Cell 126, 663–676 (2006). 16904174 10.1016/j.cell.2006.07.024

[bib36] Behr, R. & Kaestner, K. H. Developmental and cell type-specific expression of the zinc finger transcription factor Kruppel-like factor 4 (Klf4) in postnatal mouse testis. Mech. Dev. 115, 167–169 (2002). 12049784 10.1016/s0925-4773(02)00127-2

[bib37] Behr, R. et al. Kruppel-like factor 4 expression in normal and pathological human testes. Mol. Hum. Reprod. 13, 815–820 (2007). 17932114 10.1093/molehr/gam064

[bib38] Grabole, N. et al. Prdm14 promotes germline fate and naive pluripotency by repressing FGF signalling and DNA methylation. EMBO Rep. 14, 629–637 (2013). 23670199 10.1038/embor.2013.67 PMC3701237

[bib39] Watanabe, A. , Yamada, Y. & Yamanaka, S. Epigenetic regulation in pluripotent stem cells: a key to breaking the epigenetic barrier. Philos. Trans. R. Soc. Lond. B Biol. ScI. 368, 20120292 (2013). 23166402 10.1098/rstb.2012.0292 PMC3539367

[bib40] Mathieu, M. G. et al. HAGE, a cancer/testis antigen expressed at the protein level in a variety of cancers. Cancer Immun. 10, 2 (2010). 20058853 PMC2964012

[bib41] Pandey, R. R. et al. Tudor domain containing 12 (TDRD12) is essential for secondary PIWI interacting RNA biogenesis in mice. *Proc. Natl Acad. Sci. USA* 110, 16492–16497 (2013). 24067652 10.1073/pnas.1316316110 PMC3799322

[bib42] Zhou, J. , Yang, F. , Leu, N. A. & Wang, P. J. MNS1 is essential for spermiogenesis and motile ciliary functions in mice. PLoS Genet. 8, e1002516 (2012). 22396656 10.1371/journal.pgen.1002516 PMC3291534

[bib43] Maymon, B. B. et al. Localization of the germ cell-specific protein, hnRNP G-T, in testicular biopsies of azoospermic men. Acta Histochem. 104, 255–261 (2002). 12389739 10.1078/0065-1281-00657

[bib44] Magnusdottir, E. & Surani, M. A. How to make a primordial germ cell. Development 141, 245–252 (2014). 24381195 10.1242/dev.098269

[bib45] Magnusdottir, E. et al. A tripartite transcription factor network regulates primordial germ cell specification in mice. Nat. Cell Biol. 15, 905–915 (2013). 23851488 10.1038/ncb2798 PMC3796875

[bib46] Nakaki, F. et al. Induction of mouse germ-cell fate by transcription factors *in vitro*. Nature 501, 222–226 (2013). 23913270 10.1038/nature12417

[bib47] Ma, Z. , Swigut, T. , Valouev, A. , Rada-Iglesias, A. & Wysocka, J. Sequence-specific regulator Prdm14 safeguards mouse ESCs from entering extraembryonic endoderm fates. Nat. Struct. Mol. Biol. 18, 120–127 (2011). 21183938 10.1038/nsmb.2000

[bib48] Okashita, N. et al. PRDM14 promotes active DNA demethylation through the ten-eleven translocation (TET)-mediated base excision repair pathway in embryonic stem cells. Development 141, 269–280 (2014). 24335252 10.1242/dev.099622

[bib49] Chia, N. Y. et al. A genome-wide RNAi screen reveals determinants of human embryonic stem cell identity. Nature 468, 316–320 (2010). 20953172 10.1038/nature09531

[bib50] Nagamatsu, G. et al. A germ cell-specific gene, Prmt5, works in somatic cell reprogramming. J. Biol. Chem. 286, 10641–10648 (2011). 21270127 10.1074/jbc.M110.216390 PMC3060515

[bib51] Terashima, K. et al. Genome-wide analysis of DNA copy number alterations and loss of heterozygosity in intracranial germ cell tumors. Pediatr. Blood Cancer 61, 593–600 (2014). 24249158 10.1002/pbc.24833

[bib52] Ruark, E. et al. Identification of nine new susceptibility loci for testicular cancer, including variants near DAZL and PRDM14. Nat. Genet. 45, 686–689 (2013). 23666240 10.1038/ng.2635 PMC3680037

[bib53] Yamaji, M. et al. PRDM14 ensures naive pluripotency through dual regulation of signaling and epigenetic pathways in mouse embryonic stem cells. Cell Stem Cell 12, 368–382 (2013). 23333148 10.1016/j.stem.2012.12.012

[bib54] Mizuno, Y. , Gotoh, A. , Kamidono, S. & Kitazawa, S. [Establishment and characterization of a new human testicular germ cell tumor cell line (TCam-2)]. Nihon Hinyokika Gakkai Zasshi 84, 1211–1218 (1993). 8394948 10.5980/jpnjurol1989.84.1211

[bib55] de Jong, J. et al. Further characterization of the first seminoma cell line TCam-2. Genes Chromosomes Cancer 47, 185–196 (2008). 18050305 10.1002/gcc.20520

[bib56] Andrews, P. W. Teratocarcinomas and human embryology: pluripotent human EC cell lines. Review article. APMIS 106, 158–167, discussion 167-8 (1998). 9524574 10.1111/j.1699-0463.1998.tb01331.x

[bib57] Roach, S. , Schmid, W. & Pera, M. F. Hepatocytic transcription factor expression in human embryonal carcinoma and yolk sac carcinoma cell lines: expression of HNF-3 alpha in models of early endodermal cell differentiation. Exp. Cell Res. 215, 189–198 (1994). 7957668 10.1006/excr.1994.1331

[bib58] Lowe, R. & Rakyan, V. K. Marmal-aid--a database for Infinium HumanMethylation450. BMC Bioinformatics 14, 359 (2013). 24330312 10.1186/1471-2105-14-359 PMC3878775

[bib59] Heyn, H. et al. Distinct DNA methylomes of newborns and centenarians. *Proc. Natl Acad. Sci. USA* 109, 10522–10527 (2012). 22689993 10.1073/pnas.1120658109 PMC3387108

[bib60] Irizarry, R. A. et al. Exploration, normalization, and summaries of high density oligonucleotide array probe level data. Biostatistics 4, 249–264 (2003). 12925520 10.1093/biostatistics/4.2.249

[bib61] Pfaffl, M. W. A new mathematical model for relative quantification in real-time RT-PCR. Nucleic Acids Res. 29, e45 (2001). 11328886 10.1093/nar/29.9.e45 PMC55695

