# Supplementary material for: Genome-wide methylation analysis identifies genes silenced in non-seminoma cell lines
Source: NPJ Genom Med. 2016 Jan 13;1:15009–. doi: 10.1038/npjgenmed.2015.9 (PMC5685295; doi:10.1038/npjgenmed.2015.9)

NORTH SHORES

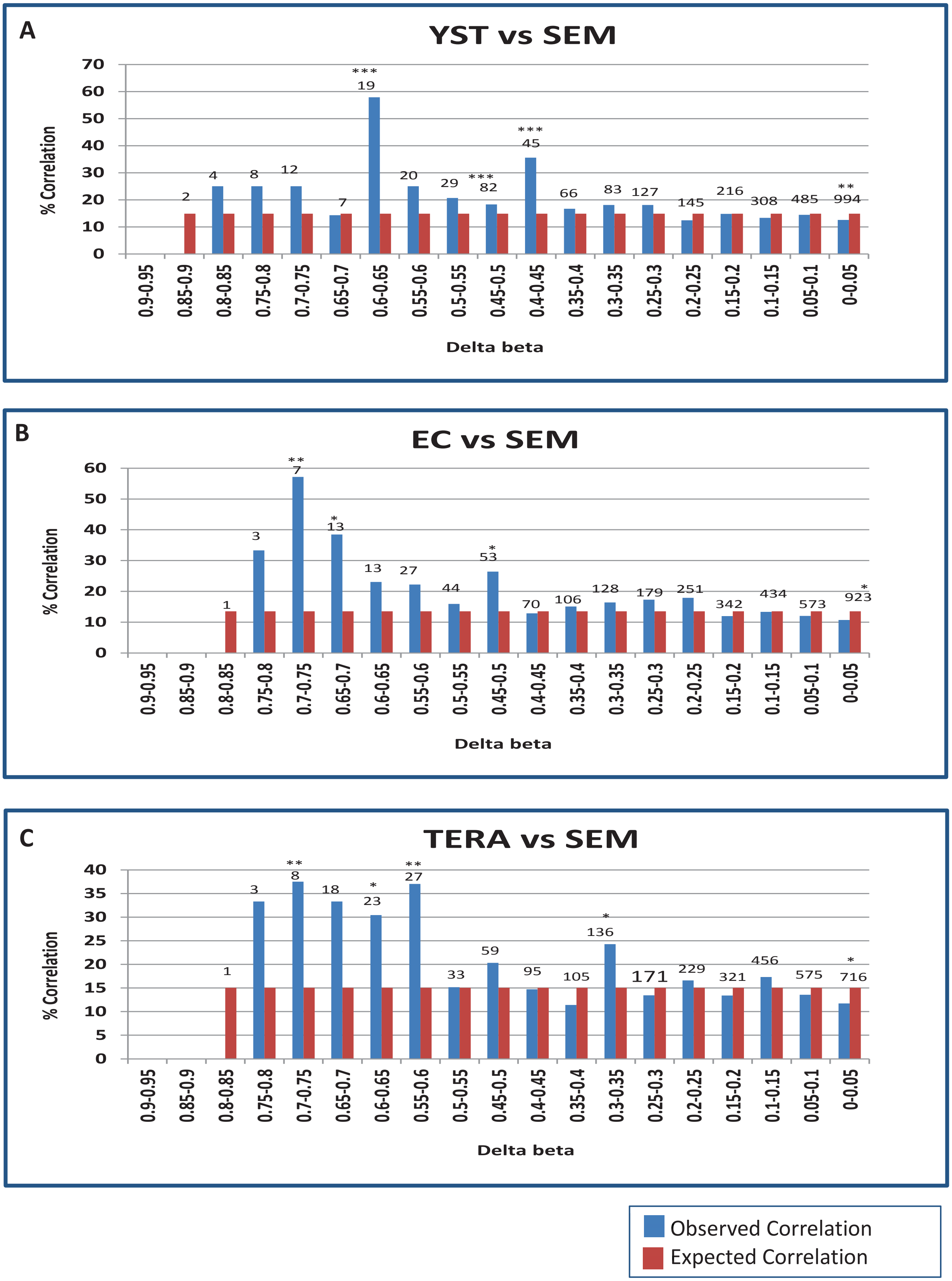

SOUTH SHORES

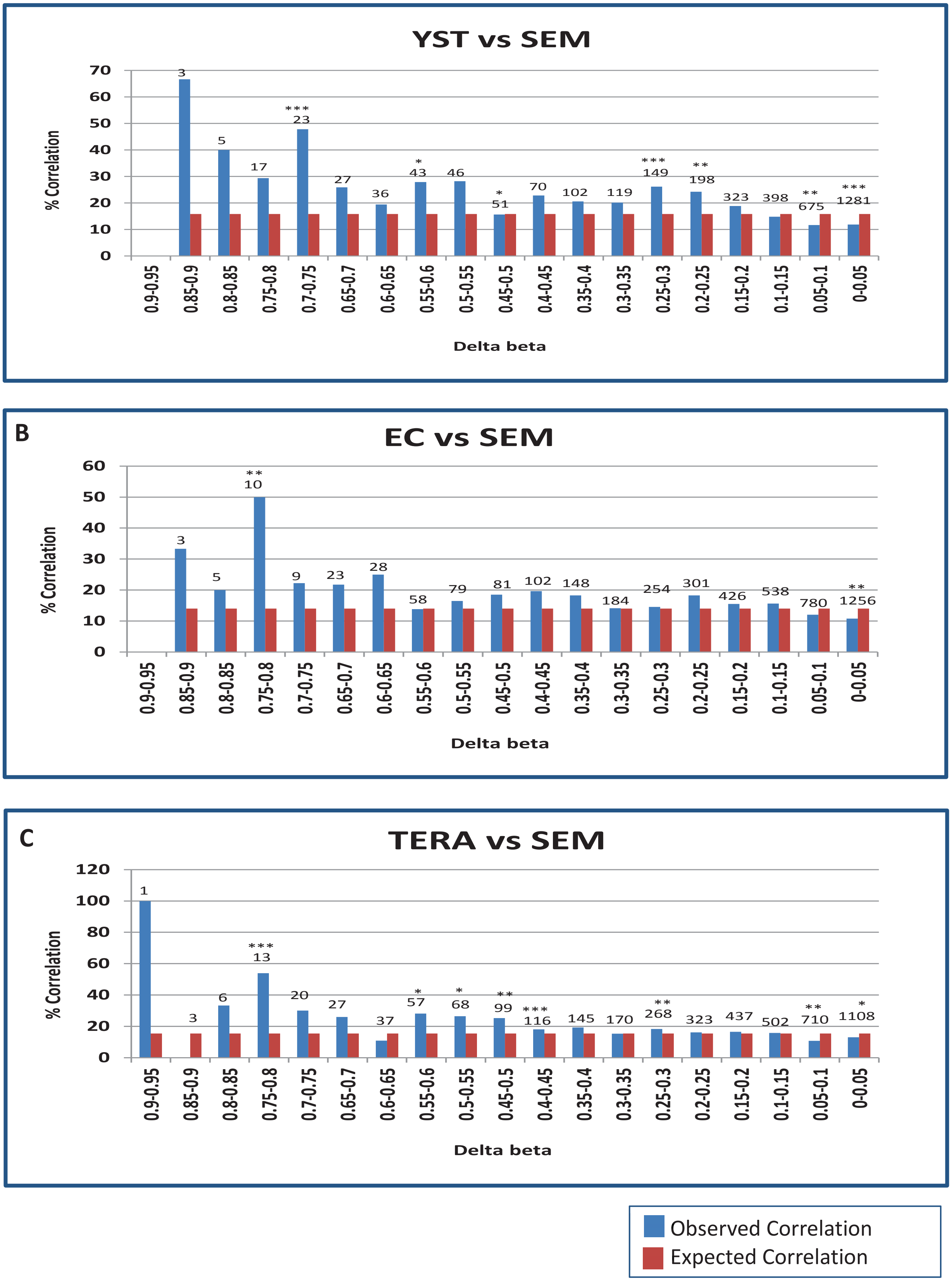

NORTH SHELVES

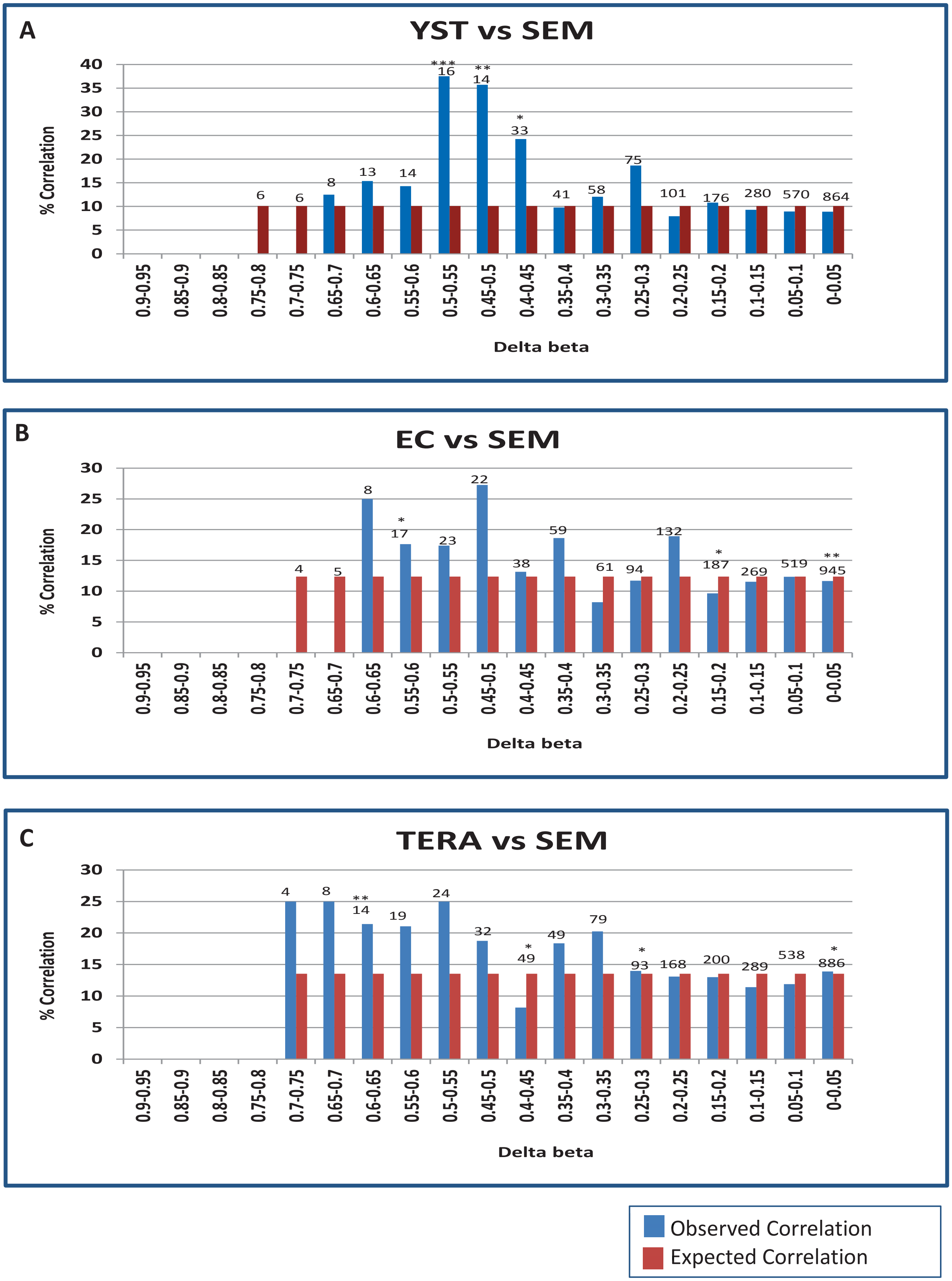

SOUTH SHELVES

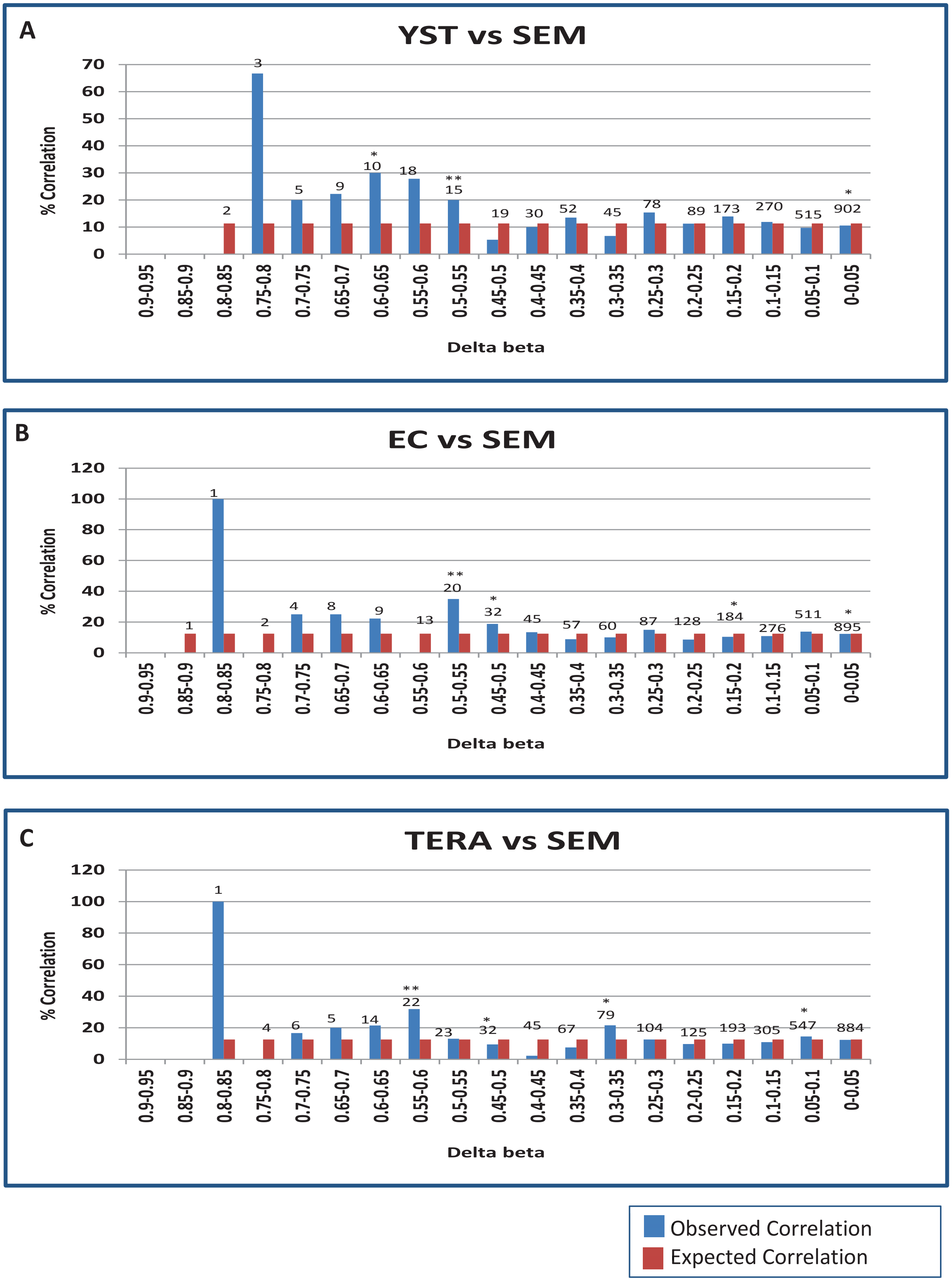

Supplement: Supplementary Figure S1 [file npjgenmed20159-s1.pdf]
