# Supplementary material for: Genome-wide methylation analysis identifies genes silenced in non-seminoma cell lines
Source: NPJ Genom Med. 2016 Jan 13;1:15009–. doi: 10.1038/npjgenmed.2015.9 (PMC5685295; doi:10.1038/npjgenmed.2015.9)
Supplement: Supplementary Figure S2 [file npjgenmed20159-s2.pdf]

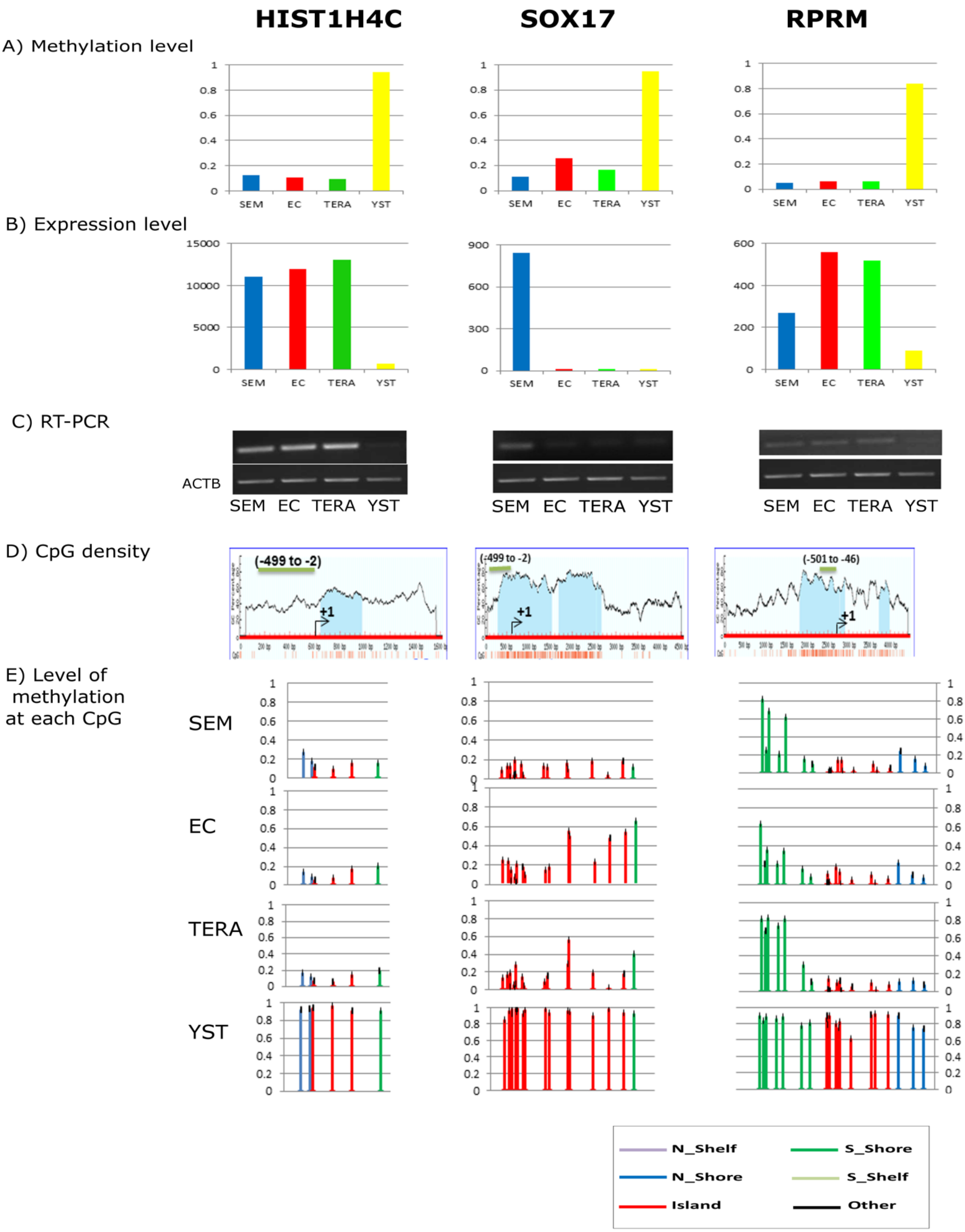
HIST1H4C
SOX17
RPRM
A) Methylation level
SEM
EC
TERA
YST
B) Expression level
C) RT-PCR
ACTB
D) CpG density
(-499 to -2)
(-501 to -46)
+1
E) Level of methylation at each CpG
N_Shelf
N_Shore
Island
S_Shore
S_Shelf
Other

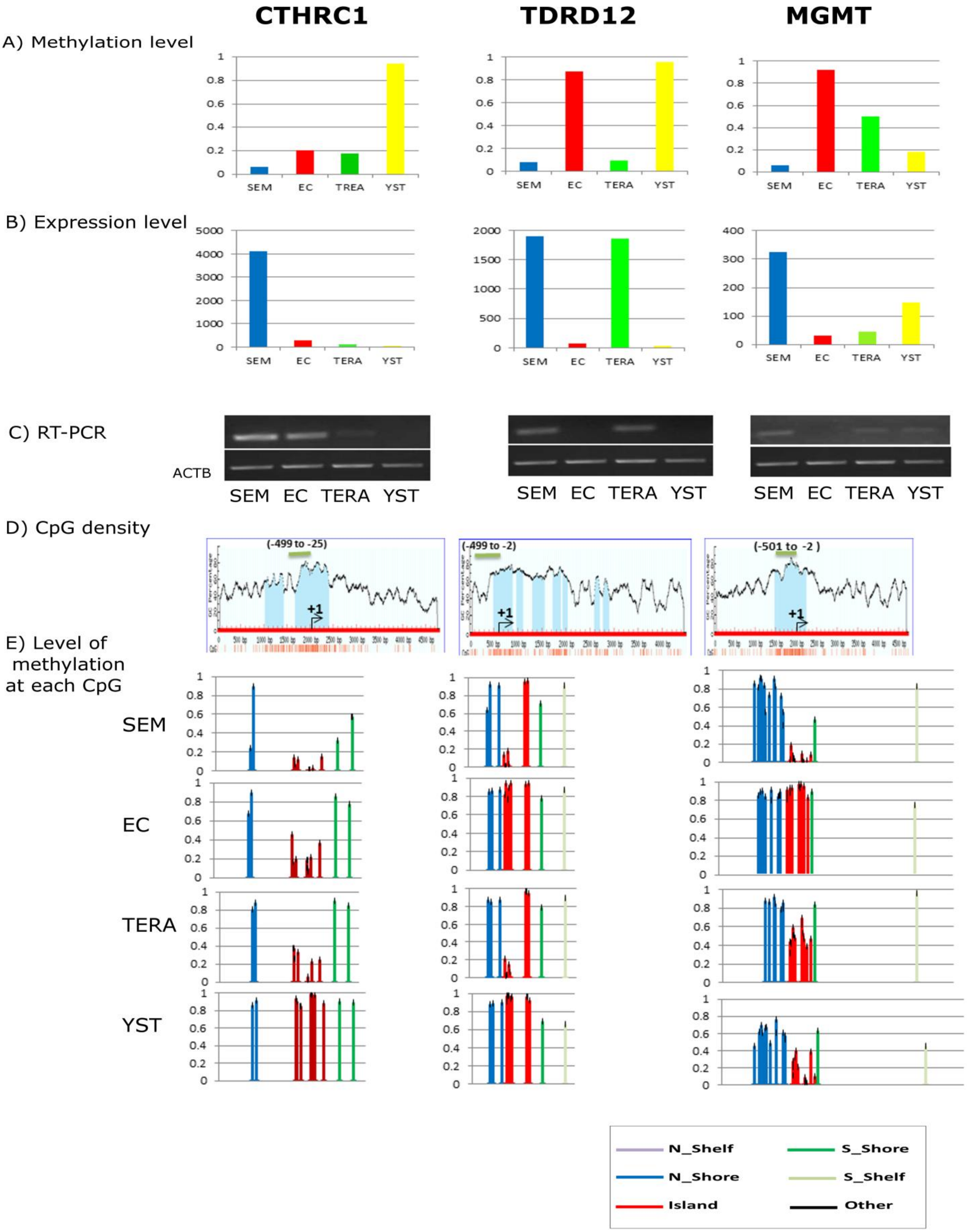

CTHRC1
TDRD12
MGMT
A) Methylation level
SEM
EC
TREA
TERA
YST
B) Expression level
C) RT-PCR
ACTB
D) CpG density
(-499 to -25)
(-499 to -2)
(-501 to -2)
+1
E) Level of methylation at each CpG
N_Shelf
N_Shore
Island
S_Shore
S_Shelf
Other

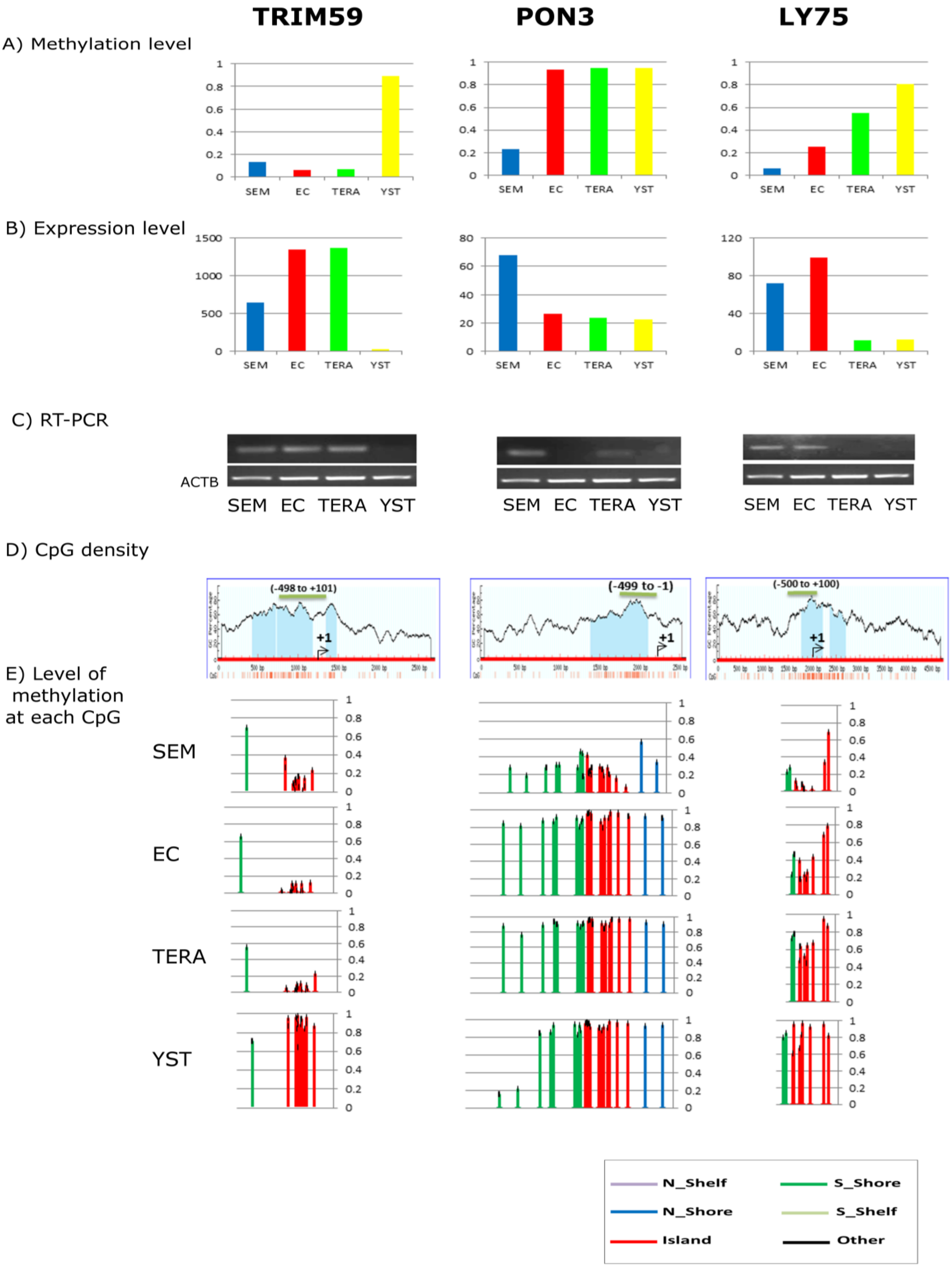

TRIM59
PON3
LY75
A) Methylation level
B) Expression level
C) RT-PCR
ACTB
SEM EC TERA YST
D) CpG density
(-498 to +101)
(-499 to -1)
(-500 to +100)
+1
E) Level of methylation at each CpG
SEM
EC
TERA
YST
N_Shelf
N_Shore
Island
S_Shore
S_Shelf
Other

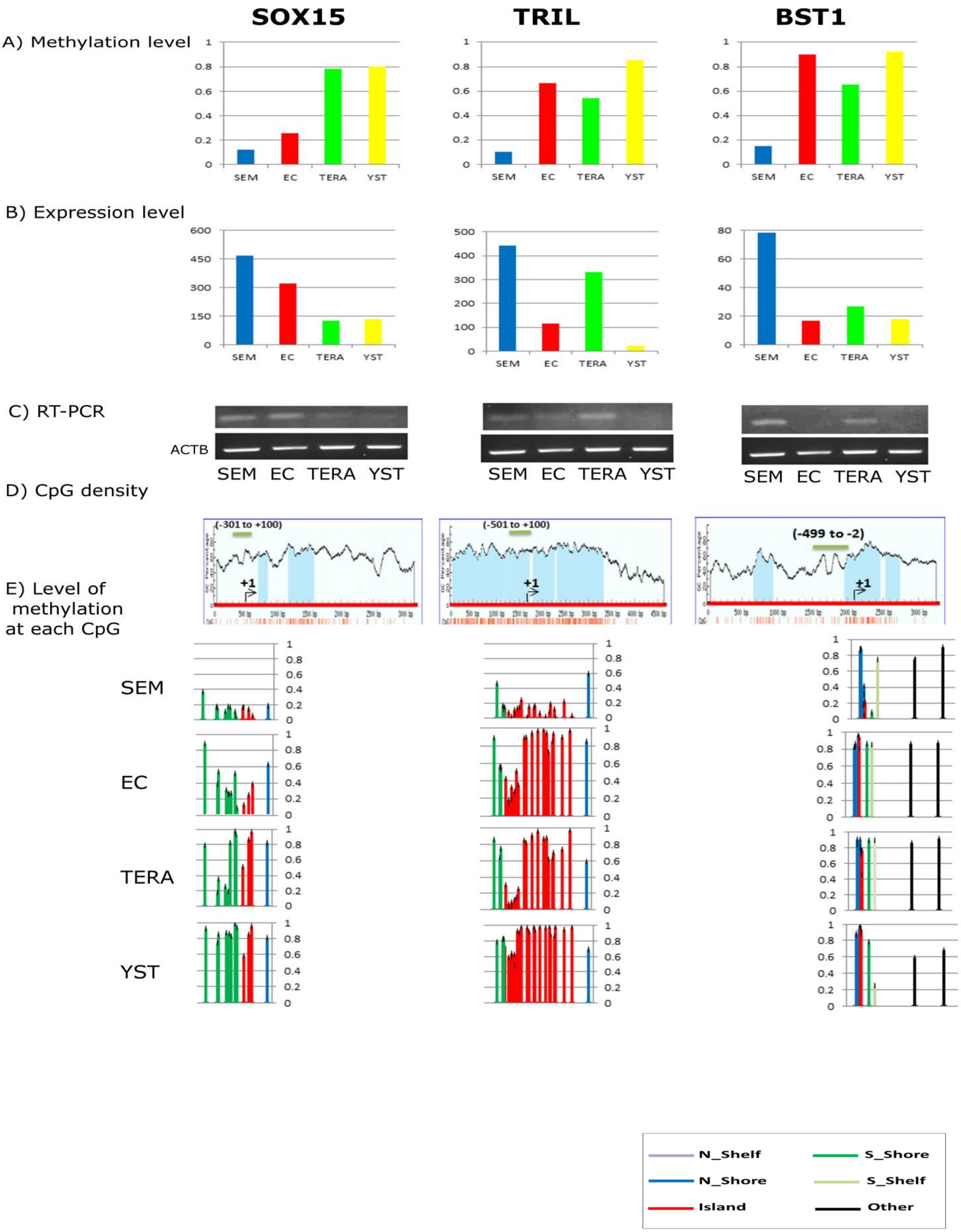

SOX15
TRIL
BST1
A) Methylation level
B) Expression level
C) RT-PCR
ACTB
SEM EC TERA YST
D) CpG density
(-301 to +100)
(-501 to +100)
(-499 to -2)
+1
E) Level of methylation at each CpG
SEM
EC
TERA
YST
N_Shelf
N_Shore
Island
S_Shore
S_Shelf
Other

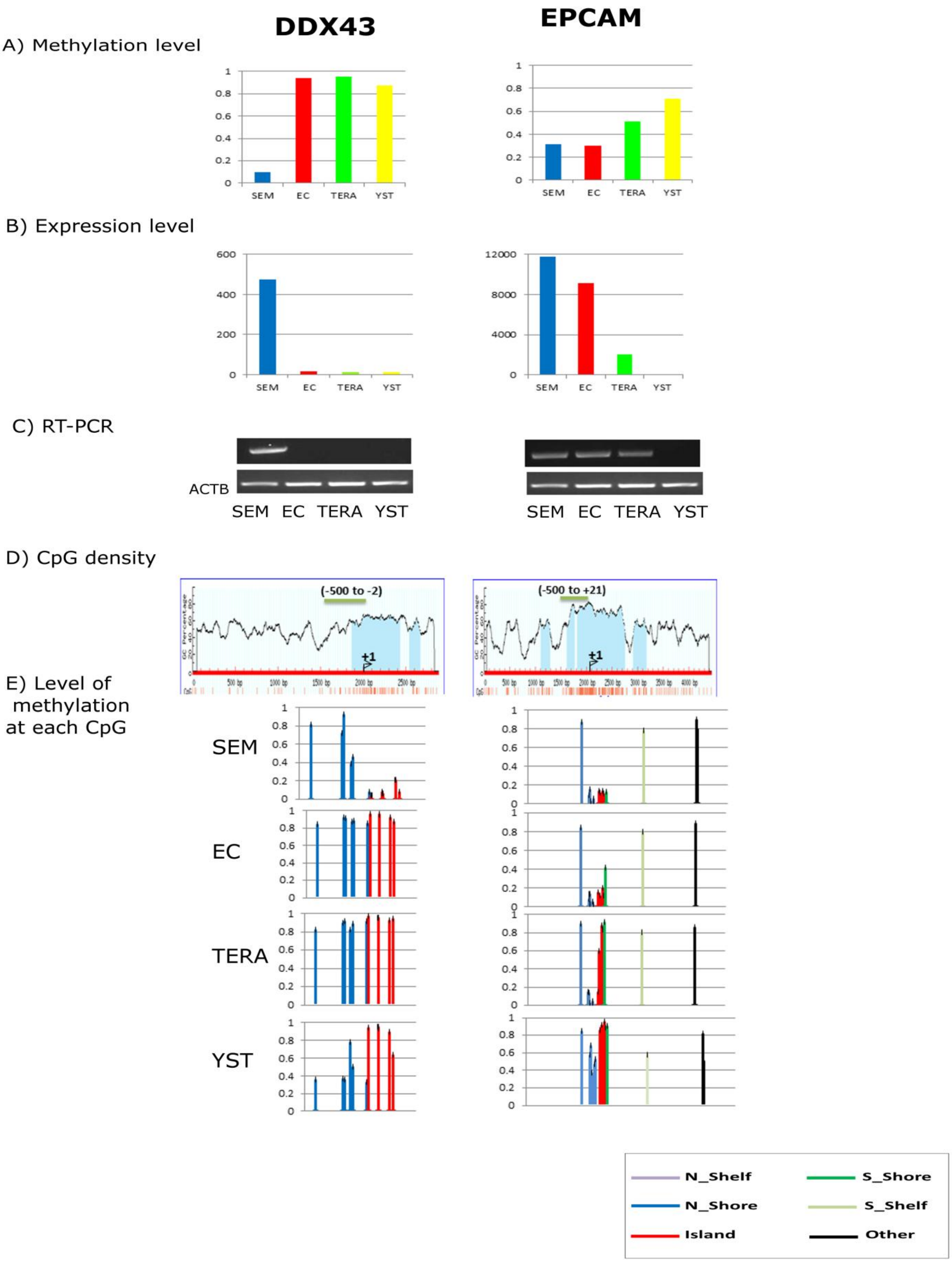

DDX43
EPCAM
A) Methylation level
B) Expression level
C) RT-PCR
ACTB
SEM EC TERA YST
D) CpG density
(-500 to -2)
(-500 to +21)
+1
E) Level of methylation at each CpG
SEM
EC
TERA
YST
N_Shelf
N_Shore
Island
S_Shore
S_Shelf
Other
