# Supplementary material for: Genome-wide methylation analysis identifies genes silenced in non-seminoma cell lines
Source: NPJ Genom Med. 2016 Jan 13;1:15009–. doi: 10.1038/npjgenmed.2015.9 (PMC5685295; doi:10.1038/npjgenmed.2015.9)
Supplement: Supplementary Figure S5 [file npjgenmed20159-s5.pdf]

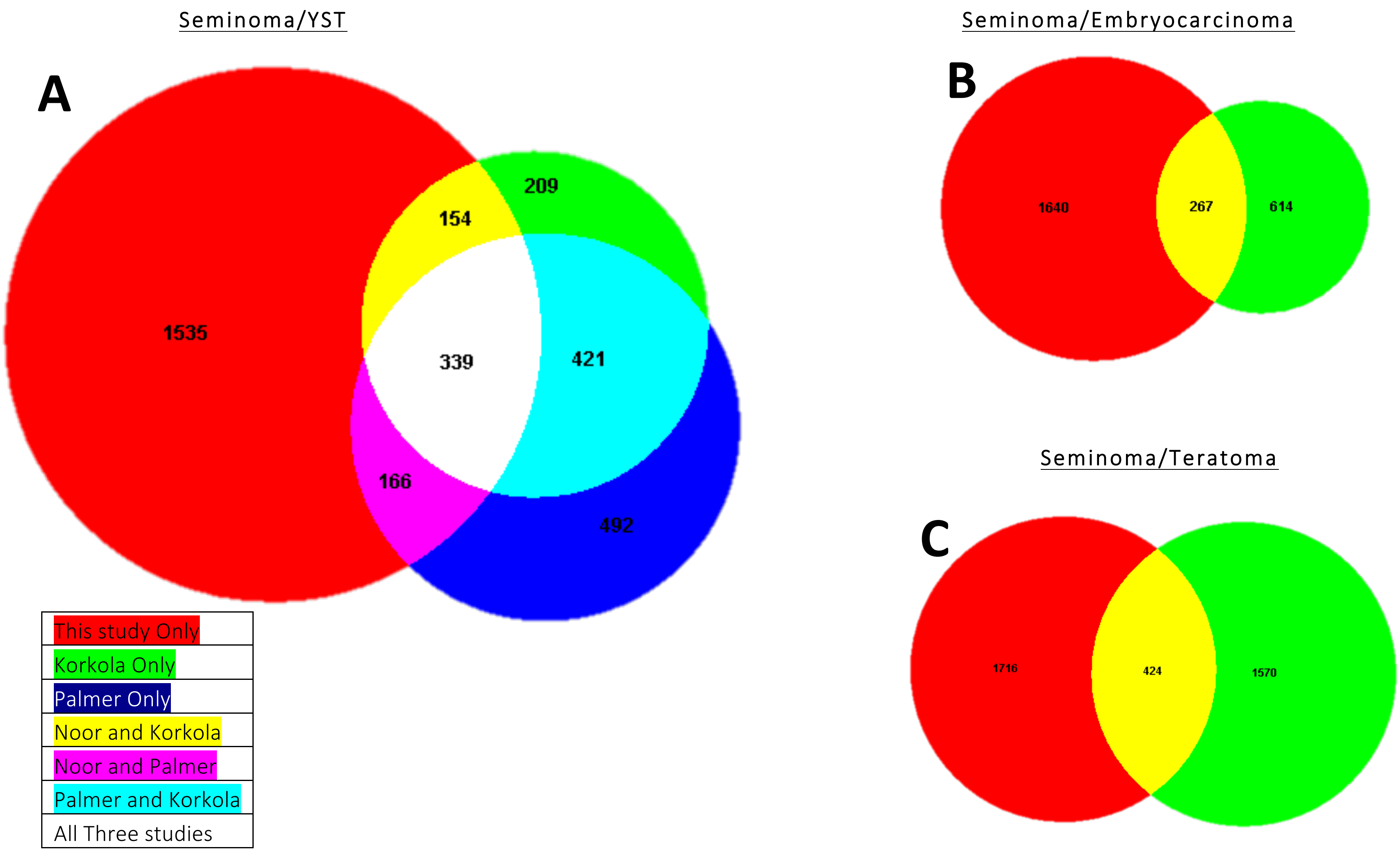
Seminoma/YST
A
1535
154
209
339
421
166
492
This study Only
Korkola Only
Palmer Only
Noor and Korkola
Noor and Palmer
Palmer and Korkola
All Three studies
Seminoma/Embryocarcinoma
B
1640
267
614
Seminoma/Teratoma
C
1716
424
1570
